# N-Linked Glycosylation Regulates CD22 Organization and Function

**DOI:** 10.3389/fimmu.2019.00699

**Published:** 2019-04-04

**Authors:** Laabiah Wasim, Fathima Hifza Mohamed Buhari, Myuran Yoganathan, Taylor Sicard, June Ereño-Orbea, Jean-Philippe Julien, Bebhinn Treanor

**Affiliations:** ^1^Department of Immunology, University of Toronto, Toronto, ON, Canada; ^2^Department of Cell and Systems Biology, University of Toronto, Toronto, ON, Canada; ^3^Department of Biological Sciences, University of Toronto Scarborough, Toronto, ON, Canada; ^4^Department of Biochemistry, University of Toronto, Toronto, ON, Canada; ^5^The Hospital for Sick Children Research Institute, Toronto, ON, Canada

**Keywords:** B cell receptor, B cell activation, signaling, CD22, glycosylation, galectin-9, super-resolution imaging

## Abstract

The organization and clustering of cell surface proteins plays a critical role in controlling receptor signaling; however, the biophysical mechanisms regulating these parameters are not well understood. Elucidating these mechanisms is highly significant to our understanding of immune function in health and disease, given the importance of B cell receptor (BCR) signaling in directing B cells to produce antibodies for the clearance of pathogens, and the potential deleterious effects of dysregulated BCR signaling, such as in B cell malignancies or autoimmune disease. One of main inhibitory co-receptors on B cells is CD22, a sialic-acid binding protein, which interacts homotypically with other sialylated CD22 molecules, as well as heterotypically with IgM and CD45. Although the importance of CD22 in attenuating BCR signaling is well established, we still do not fully understand what mediates CD22 organization and association to BCRs. CD22 is highly glycosylated, containing 12 N-linked glycosylation sites on its extracellular domain, the function of which remain to be resolved. We were interested in how these glycosylation sites mediate homotypic vs. heterotypic interactions. To this end, we mutated five out of the six N-linked glycosylation residues on CD22 localized closest to the sialic acid binding site. Glycan site N101 was not mutated as this resulted in lack of CD22 expression. We used dual-color super-resolution imaging to investigate the impact of altered glycosylation of CD22 on the nanoscale organization of CD22 and its association with BCR. We show that mutation of these five glycosylation sites increased the clustering tendency of CD22 and resulted in higher density CD22 nanoclusters. Consistent with these findings of altered CD22 organization, we found that mutation of N-glycan sites attenuated CD22 phosphorylation upon BCR stimulation, and consequently, increased BCR signaling. Importantly, we identified that these sites may be ligands for the soluble secreted lectin, galectin-9, and are necessary for galectin-9 mediated inhibition of BCR signaling. Taken together, these findings implicate N-linked glycosylation in the organization and function of CD22, likely through regulating heterotypic interactions between CD22 and its binding partners.

## Introduction

B cells drive the humoral immune response against extracellular pathogen by recognizing antigen through their B cell receptor (BCR). Specific binding of cognate antigen to BCR initiates the process of B cell activation as antigen-BCR complexes are internalized, processed, and subsequently antigen-derived peptides are presented in the context of major histocompatibility (MHC) class II on the B cell surface for T cell help. Efficient B cell activation ultimately leads to B cell proliferation and differentiation into high-affinity antibody producing plasma cells and memory B cells that provide an accelerated response during secondary exposure (1).

In order for B cells to carry out effector functions against a wide range of pathogens, B cell development must generate a highly diverse set of antigen receptor specificities. Through the process of somatic recombination, germline encoded gene fragments are stochastically rearranged to produce functional BCRs. The randomness of this process generates highly diverse specificities; however, many turn out to be self-reactive (2). It is estimated that up to 70% of the immature human B cell repertoire is autoreactive and up to 30–40% of human peripheral B cells recognize self-antigens (3). Genome-wide association studies reveal autoimmunity-associated variants are highly enriched for genes that affect B cell signaling, including genes that encode receptors, signaling effectors and downstream transcriptional regulators of the BCR (4). This is evident in autoimmune diseases such as systemic lupus erythematosus (SLE) and rheumatoid arthritis, where chronic B cell activation and high levels of auto-antibody production leads to systemic inflammation and tissue damage (5). Early events of antigen recognition define the strength of B cell signaling, and therefore, fine-tuning of B cell activation threshold in an antigen dependent context is of utmost importance in both maintaining B cell tolerance and mounting an efficient immune response.

Recently, we identified galectin-9, a β-galactoside-binding protein, as crucial in regulating BCR signaling and activation (6). Galectin-9 is a member of the tandem-repeat subfamily of galectins, containing two carbohydrate-recognition domains (CRDs) connected by a flexible linker (7). Galectins have been implicated in the formation of lattice-like structures through binding of different glycoproteins and glycolipids on the extracellular surface of cells in order to regulate a wide range of functions such as protein trafficking, cell migration, signaling, and apoptosis (8). We identified IgM-BCR and inhibitory receptor CD45 as ligands for galectin-9, and demonstrated that galectin-9 inhibits antigen-BCR microcluster formation upon BCR stimulation, concomitant with suppression of downstream BCR signaling. The mechanism for galectin-9 regulation of BCR signaling may involve the inhibitory co-receptor CD22, however the requirement of CD22 in galectin-9 mediated inhibition of BCR signaling has not been demonstrated.

CD22 is a member of the sialic acid-binding immunoglobulin-like lectin (Siglec) receptor family (9, 10). It is a B cell-lineage-restricted receptor bearing three immunoreceptor tyrosine-based inhibitory motifs (ITIMs) on its cytoplasmic tail and is crucial in preventing abnormal B cell activation (11). Consequently, CD22 deficiencies, and genetic variants are associated with hyperactive B cells and have been implicated in autoimmune disease (12–14). The main ligand for CD22 seems to be CD22 itself, forming oligomers on the surface of B cells via homotypic binding (15). Recent structural analysis of CD22 demonstrated that the ectodomain of CD22 adopts an elongated but bent, low flexibility conformation, hypothesized to allow homotypic interactions in *cis* and the formation of CD22 nanoclusters (16). CD22 has also been shown to interact with IgM-BCR and the phosphatase CD45 by immunoprecipitation assays (17–22). In the resting state, only a portion of CD22 is associated with BCR (23); however, upon B cell activation association of CD22 with IgM-BCR is increased (24). Interestingly, mutation of the sialic acid binding site of CD22, or treatment with sialidase, does not disrupt the interaction between CD22 and IgM-BCR or CD45, implying alternate mechanisms independent of direct CD22 sialic acid binding (22).

Given the importance of CD22 in attenuating BCR signaling, we wanted to further understand what mediates CD22 organization and association to IgM-BCRs. CD22 contains 12 N-linked glycosylation sites in its extracellular domain. Six glycosylation sites are located in the first two domains of CD22 and in close proximity to the sialic acid binding site (16), the function of which remain to be resolved. Thus, we investigated the role of these glycosylation sites in the organization and function of CD22 in attenuating BCR signaling. We found that mutation of five of these N-glycan sites increased the density of CD22 nanoclusters, decreased CD22 phosphorylation upon BCR stimulation, and consequently enhanced B cell signaling. We also identified an important role for these sites in galectin-9 mediated inhibition of BCR signaling and CD22-IgM association, and propose that one of these sites may be a direct ligand of galectin-9. These findings have important implications for our understanding of the role of CD22 in maintaining self-tolerance, and the potential dysfunction of CD22 in the context of autoimmune diseases. Moreover, our findings highlight the potential for therapeutic use of galectin-9 in the treatment of autoimmune diseases.

## Materials and Methods

### Cell Lines and Culturing

Daudi B cells were maintained at 37°C with 5% CO_2_ in RPMI 1640 containing 10% heat-inactivated fetal bovine serum (FBS), 100 U/mL penicillin and streptomycin (Gibco), and 50 μM 2-mercaptoethanol (Amresco). Parental Daudi B cells and CD22-KO Daudi B cells were kindly provided by Dr. Joan Wither (Krembil Research Institute, Toronto).

### Stable Transfection of CD22 Constructs

CD22-KO Daudi B cells were transfected with 10 μg of WT human CD22β plasmid or 5Q human CD22β plasmid, containing point mutations from asparagine to glutamine at N67, N112, N135, N164, and N231, thereby abrogating N-linked glycosylation at that site. Plasmid DNA was electroporated into cells using Gene Pulser Xcell (Bio-Rad) at 570 V, 25 μFD. Positive populations were sorted by 0.8 mg/ml Geneticin (Thermo Fisher®) for 30 days followed by FACS sorting of positive population labeled with humanized anti-CD22 Fab fragment [pinatuzumab (16)] at 5 μg/ml.

### Mice

C57BL/6 (Wildtype; WT) mice were obtained from Charles River, *CD22*^−/−^ (CD22-KO) mice were obtained from Dr. Cynthia Guidos (Sick Kids Research Institute). Mice were housed in specific pathogen-free animal facility at University of Toronto Scarborough, or Sick Kids Research Instutite, Toronto, Canada. All procedures were approved by the Local Animal Care Committee (LACC) at the University of Toronto Scarborough. Splenocytes were isolated from 2 to 4 month old mice and were sex matched within each experiment. B cells were purified by magnetic negative selection according to the manufacturer's protocol (Stem Cell Technologies).

### Antibody and Fab Fragment Labeling for Flow Cytometry and Imaging

Humanized anti-CD22 Fab fragment and full-length antibody [pinatuzumab (16)] and goat anti-human IgM Fab fragment (Jackson ImmunoResearch Laboratories) were labeled at 1 mg/ml in 0.1 M NaHCO_3_ and incubated with 0.2 mg/ml Alexa Fluor® 647 NHS Ester and Alexa Fluor® 488 NHS Ester (Thermo Fisher), respectively for 1 h at room temperature. For single particle tracking, pinatuzumab and goat anti-human IgM Fab fragments (Jackson ImmunoResearch Laboratories) were labeled at 1 mg/ml in 0.1 M NaHCO_3_ and incubated with 40 μg/ml Attotec® 633 NHS Ester and Attotec® 488 NHS Ester, respectively for 1 h at room temperature with gentle mixing. Following labeling, the mixture was dialyzed against PBS at 4°C. After changing the buffer two times in 24 h, labeled antibody was collected and stored at 4°C.

### Cell Surface Staining for CD22 by Flow Cytometry

Daudi B cells were washed with FACS buffer (1% BSA, 0.01% NaN_3_ in PBS), then immunostained with Alexa Fluor® 647-conjugated pinatuzumab Fab fragment at 1:200 v/v for 30 min at 4°C. Cells were washed and resuspended in FACS buffer for flow analysis.

### Recombinant Gal9 (rGal9) Treatment and Staining

B cells were treated with 1 μM recombinant galectin-9 (R&D Systems) in 2% BSA in RPMI for 30 min at 37°C. Cells were washed 1X with PBS or RPMI and resuspended in PBS for immediate use in experiments. To stain for rGal9, Daudi cells were first blocked with purified anti-human CD16/CD32 (Fc-block, 1:250 v/v; BD Biosciences, Cat. No 564220) in PBS for 15 min at room temperature. Cells were washed two times and immunostained with goat anti-galectin-9 antibody (R&D Systems, Cat. No. AF3535) at 1:100 v/v for 1 h in 2% BSA in PBS. Cells were washed 3 times, followed by labeling with Cy3-conjugated AffiniPure bovine anti-goat antibody (Jackson Immunoresearch Laboratories, Cat. No. 805-165-180) at 1:1000 v/v in 2% BSA in RPMI for 30 min at 4°C.

### Cell Stimulation and Western Blot

Daudi cells were resuspended at 2 × 10^6^ cells/ml in RPMI 1640 and stimulated for the indicated time with 5 μg/ml AffiniPure F(ab')_2_ goat anti-human IgM, μ-chain specific (Jackson ImmunoResearch Laboratories) or 20 μg/ml anti-CD22 mAb (Epratuzumab). Primary murine B cells from WT and CD22-KO mice treated or not with rGal9 as above were resuspended at 3 × 10^6^ cells and stimulated for the indicated time with 5 μg/ml AffiniPure F(ab')_2_ goat anti-mouse IgM, μ-chain specific (Jackson ImmunoResearch Laboratories). The reaction was stopped by adding 1 ml of ice cold PBS. Cells were centrifuged at 15,000 × g for 30 s. Supernatant was removed and lysis buffer (1% NP40, 0.15 M NaCl, 20 nM Tris pH 8.9, 10 mM NaF, 1 mM Na_3_VO_4_ and Roche cOmplete^TM^ protease inhibitor cocktail) was added at 10 × 10^7^ cells/ml. Cells were incubated at 4°C with gentle mixing for 30 min. Cell lysate was then centrifuged at 15,000 × g for 15 min to remove cellular debris and the supernatant was transferred to a clean microtube. 2X Laemmli buffer containing 0.1 M DTT was added to cell lysate. Samples were boiled at 95°C for 5 min followed by SDS-PAGE. Proteins were transferred to a PVDF membrane and blocked in 5% BSA in TBS-T (20 mM Tris pH 7.5, 150 mM NaCl and 0.1% Tween 20) for 1 h or overnight at 4°C. Membranes were immunoblotted with the following antibodies in 1% BSA/TBST for 5 h at room temperature or overnight at 4°C with gentle rocking: mouse anti-β-tubulin (Sigma, 1:5,000 v/v), rabbit anti-phospho CD22 Y807 (Abcam, 1:2,000 v/v), rabbit anti-phospho CD22 Y842 (Abcam, 1:2,000 v/v), mouse anti-phospho CD22 Y822 (BD Biosciences, 1:200 v/v), rabbit anti-phospho CD19 Y531 (Cell Signaling Technology, 1:1,000 v/v), rabbit anti-phospho CD79a Y182 (Cell Signaling Technology, 1:1,1000 v/v), PLCγ2 Y1217 (Cell Signaling Technology, 1:1,000 v/v), rabbit anti-phospho Akt S473 (Cell Signaling Technology, 1:1,000 v/v), and rabbit anti-phospho ERK p44/p42 MAPK (Cell Signaling Technology, 1:1,000 v/v). Membranes were washed 3 times with TBST, then incubated with HRP-conjugated donkey anti-rabbit IgG or donkey anti-mouse IgG antibodies (Jackson ImmunoResearch, 1:10,000 v/v) in 1% BSA/TBST for 1 h at room temperature. Membranes were washed 5 times with TBST, then incubated with Pierce® ECL Western Blotting Substrate or Clarity ECL (Biorad) and imaged with ChemiDoc System (Bio-Rad). The intensity of each protein band was analyzed by ImageJ, normalized to β-tubulin.

### Calcium Signaling

Daudi B cells were labeled with 1 μM Fluo-4, AM (Thermo Fisher) at a concentration of 1 × 10^6^ cells/ml in 10% FBS/HBSS at 37°C for 30 min. Cells were washed with HBSS two times and resuspended in RPMI 1640. Intracellular calcium flux was measured by flow cytometry. After collecting a baseline for 30 s, cells were stimulated with 0.5, 5, or 20 μg/ml AffiniPure F(ab')_2_ goat anti-human IgM, μ-chain specific (Jackson ImmunoResearch Laboratories). Change in fluorescence intensity was recorded and plotted using FlowJo (TreeStar). Fold change was determined by dividing florescence intensity at each time point by the baseline intensity.

### Glass Coverslip Coating for dSTORM and Single Particle Tracking

Glass coverslips were cleaned in sulfochromic acid (Thermo Fisher) for 20 min, and then rinsed in water followed by acetone. Coverslips were air-dried and then incubated with 0.2 μg/ml fibronectin (Sigma-Aldrich) for 1 h at room temperature and then washed with PBS. Coverslips were assembled into FCS2 chambers (Bioptechs).

### Single Particle Tracking

Daudi B cells were washed once in PBS then stained in 2% FBS/PBS at a concentration of 1 × 10^6^ cells/ml with 4 ng/ml of Attotec® 488 labeled goat anti-human IgM Fab fragment and 1 μg/ml of Attotec® 633 labeled pinatuzumab Fab fragment for 20 min at 4°C. Cells were washed two times with PBS and resuspended in chamber buffer (0.5% FBS, 2 mM MgCl_2_, 0.5 mM CaCl_2_, and 1 g/L D -glucose in PBS). Cells were incubated at 37°C for 5 min before injecting in FSC2 chambers preheated to 37°C. Single particle tracking (SPT) was performed on a total internal reflection fluorescence (TIRF) microscope (Quorum Technologies) based on an inverted microscope (DMI6000C, Leica), HCX PL APO 100X/1.47 oil immersion objective and Evolve Delta EMCCD camera (Photometrics). Cells were allowed to settle in chambers for 1 min prior to image acquisition and images were taken up to 10 min. Images were acquired continuously at 20 frames/s for 10 s with an EM Gain of 200 and an exposure time of 50 ms. SPT analysis was performed on a custom MATLAB script as described previously (25).

### dSTORM of CD22 Nanoclusters

#### Sample Preparation

Daudi B cells were washed in PBS once then stained in 2% FBS/PBS at a concentration of 1 × 10^6^ cells/ml with 10 μg/ml of Alexa Fluor® 647 labeled pinatuzumab IgG and 2 μg/ml Alexa Fluor® 488 labeled anti-human IgM Fab fragment for 20 min at 4°C. Cells were washed 2 times with PBS and resuspended in PBS. Cells were incubated at 37°C for 5 min before injecting in FSC2 chambers preheated to 37°C and allowed to spread for 10 min. Chambers were gently flushed with PBS to wash unbound cells and cells were fixed with 4% PFA and 0.2% glutaraldehyde in PBS for 40 min at room temperature. Chambers were washed 3 times with PBS. Prior to image acquisition, chambers were incubated with dSTORM imaging buffer containing 0.1 M β-mercaptoethylamine (MEA, Sigma-Aldrich), 0.5 mg/ml glucose oxidase, 40 μg/ml catalase, and 10% glucose in PBS. For dual-color dSTORM, samples were incubated in PBS containing 0.1 M MEA, 3% (v/v) OxyFlourTM (Oxyrase Inc.), 20% (v/v) of sodium DL-lactase solution (L1375, Sigma-Aldrich) adjusted to pH ~8.3. Fiducial markers (100 nm Tetraspeck Fluorescent Microspheres, Invitrogen) were added to buffer and allowed to settle for 5 min prior to imaging.

#### Image Acquisition and Reconstruction

dSTORM was performed on a TIRF microscope (Quorum Technologies) based on an inverted microscope (DMI6000C, Leica), HCX PL APO 100X/1.47 oil immersion objective and Evolve Delta EMCCD camera (Photometrics). For Alexa® 647, photoconversion was achieved with 633 nm laser (intensity ranged from 80 to 100 mW/cm^2^) illumination and simultaneous illumination with the 405 nm laser (intensity range from 5 to 20 mW/cm^2^) increased the rate of conversion from the dark state. Dual-color dSTORM images were acquired sequentially, first imaging in the 647-channel followed by imaging in the 488-channel. For Alexa® 488, photoconversion was achieved with the 488-nm laser (intensity ranged from 80 to 100 mW/cm^2^). Eight thousand images were acquired at 30 ms exposure with a frame rate of 33 frames/s, and EM gain of 200. Fiducial markers, which were visible in both the 488 and the 647-nm channels, were used to align the two channels prior to image reconstruction. The images of the beads in both channels were used to calculate a polynomial transformation function that mapped the 488-nm channel onto the 647-nm channel, using MultiStackReg plug-in of ImageJ. The transformation matrix was applied to each frame of the 488-nm channel stack. Image reconstruction was performed using ThunderSTORM (26) plugin for ImageJ according to the parameters previously reported (6). The camera setup was as follows: pixel size 101.5 nm, photoelectron per A/D count 2.4, base level [A/D count] 414 and an EM gain of 200. Image filtering was applied to remove camera noise and enhance photoswitching events using a wavelet filter (B-Spline) with a B-Spline order of 3 and B-Spline scale of 2.0. Approximate localization of molecules was detected by local maximum method with a peak intensity threshold of std (Wave.F1) and a connectivity of 8-neighborhood. Sub-pixel localization of molecules was identified by fitting the point spread function to an integrated Gaussian using weighted least squares method with a fitting radius of 3 pixels. Single molecules may not be adequately resolved in spatially dense organizations and thereby multiple activated molecules are detected as a single blob. Multiple emitter fitting analysis (MFA) was used to estimate the number of molecules detected as a single blob with maximum 5 molecules per fitting region. To improve the multi-emitter fitting algorithm, molecules are fitted assuming the same intensity. Super resolution images were rendered with a pixel size of 20 nm.

#### Post-processing and Image Analysis

Reconstructed dSTORM images were post-processed with drift correction using the built-in method in the ThunderSTORM plugin. The workflow for post-processing steps is as previously reported (6) and consisted of the following steps: 1. Remove duplicates, in which repeated localizations of single molecules in one frame, which may occur when using multiple-emitter, were removed based on uncertainty radius of localization; 2. Filter, in which localizations with an uncertainty >20 nm were eliminated; 3. Density filter, in which isolated localizations were removed based on the parameter of at least two neighbors are required in 50 nm radius for a localization to be accepted; 4. Drift correction, in which image drift was corrected using fiducial markers; and 5. Merging, in which molecules that appeared within 20 nm in multiple frames were merged together. For each reconstructed image a 3 × 3 μm area was selected in the middle of the cell to conduct cluster analysis. The Hopkins index and Ripley's H function analysis were performed by SuperCluster, an analysis tool kindly provided by the University of New Mexico's Spatio Temporal Modeling Center (http://stmc.unm.edu/).

#### Coordinate Based Co-Localization Analysis

Colocalization of dual dSTORM data was conducted using coordinate-based colocalization (CBC) (27) analysis using ThunderSTORM. Briefly, this method uses coordinate information of each molecule instead of an intensity-based approach, which would depend on the reconstruction and post-processing parameters chosen. Furthermore, this method takes into account the spatial distribution of each set of localizations to prevent false positive colocalization values that result when one of the molecular species is randomly organized. First, the spatial distribution function of the neighboring localizations from the same species in each channel are calculated. Then, from the individual distribution functions, a correlation coefficient is calculated and weighted by distance to the nearest neighbor of the localization's respective species. As a result, each single molecule of each species is attributed an individual colocalization value, which provides information on the molecule's local environment. CBC algorithm was applied to x-y coordinate lists of localizations in the 3 × 3 μm region of 647 and 488 channels. A search radius of 80 nm, based on the radius of IgM nanoclusters, was used to calculate degree of colocalization values varying from −1 (perfectly excluded) to +1 (perfectly co-localized).

### Statistical Analysis

Statistical analysis was performed using GraphPad Prism. The distribution of data was tested using D'Agostino-Pearson omnibus normality test. Comparisons between two groups were performed using Student's *t*-test for data with normal distribution and Mann-Whitney for data with non-normal distribution. Comparisons between multiple groups were performed by Kruskal-Wallis test for data with non-normal distribution, followed by Dunn's multiple comparisons test.

## Results

### CD22 Is Necessary for Galectin-9 Mediated Inhibition of BCR Signaling in Human Daudi B Cells

**Figure 1:**
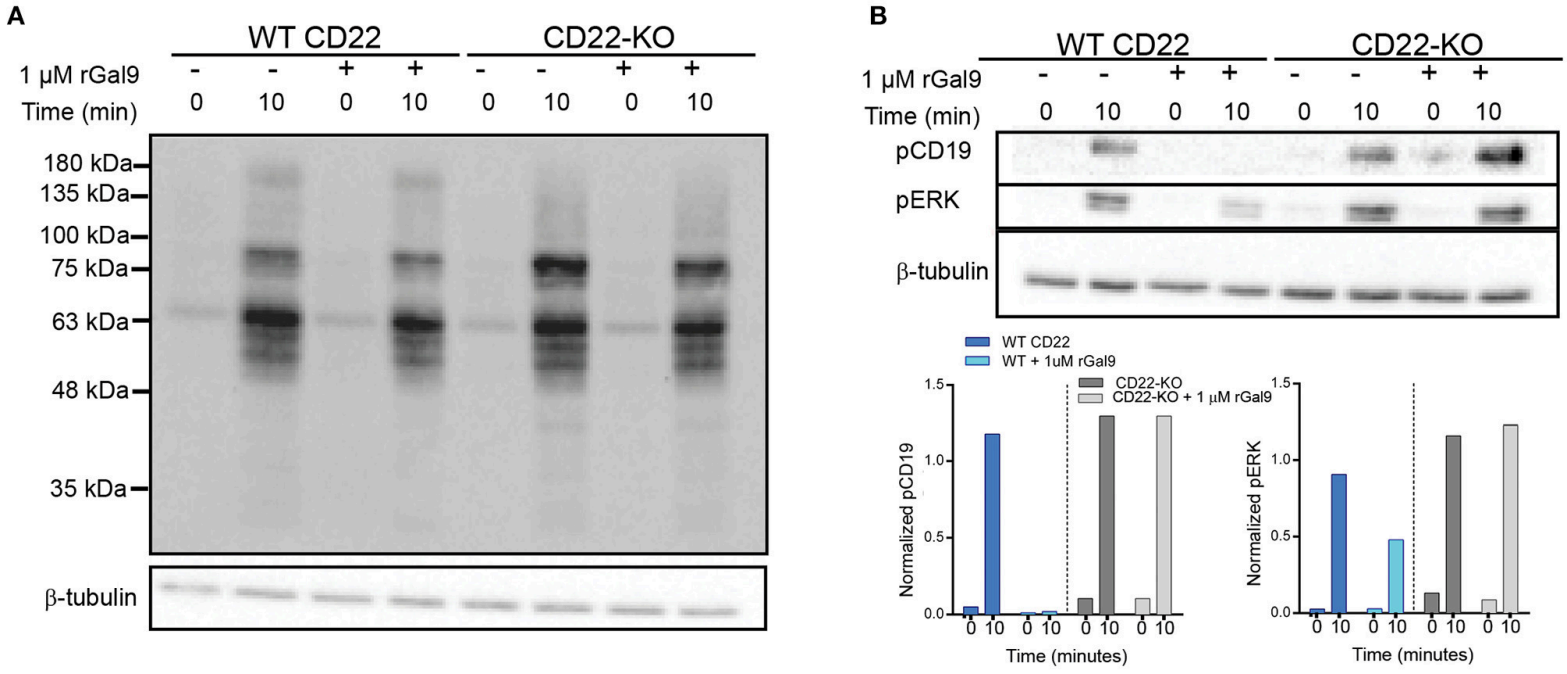
CD22 is necessary for galectin-9 mediated inhibition of BCR signaling in human Daudi B cells. WT and CD22-KO Daudi B cells treated with rGal9 were stimulated with 5 μg/ml anti-IgM F(ab')_2_ for indicated times, lysed, and subjected to SDS-PAGE followed by immunoblotting with **(A)** anti-phosphotyrosine and **(B)** phospho-CD19 and phospho-ERK. Quantifications (shown below) normalized to β-tubulin loading control. Blots and quantification representative of at least three independent experiments.

We recently demonstrated using dual-color direct stochastic optical reconstruction microscopy (dSTORM) that CD22 is endogenously associated with IgM via galectin-9 in the steady-state in primary murine B cells (6). We also found that treatment with recombinant galectin-9 increased co-localization of IgM and CD22 and suppressed BCR signaling upon antigen stimulation in primary murine B cells. To investigate if this inhibitory effect was indeed due to CD22, we utilized a human Daudi CD22-deficient B cell line generated through CRISPR-Cas9 (kindly provided by Dr. Joan Wither, Krembil Research Institute). We treated wild-type (WT) and CD22-KO B cells with recombinant galectin-9 (rGal9), stimulated cells with anti-IgM F(ab')_2_ for the indicated times and then lysed cells and ran SDS-PAGE followed by immunoblotting for total phosphotyrosine, as well as phosphorylated CD19 and extracellular regulated kinase (ERK) (Figure 1). In contrast to WT cells where treatment with rGal9 depressed total tyrosine phosphorylation, CD22-deficient cells showed a similar level of total tyrosine phosphorylation upon rGal9 addition (Figure 1A). Consistent with depressed total tyrosine phosphorylation, we found that phosphorylation of CD19 and ERK was not diminished in CD22-deficient cells upon treatment with rGal9 (Figure 1B). These findings demonstrate that galectin-9 mediated suppression of BCR signaling in human Daudi cells is dependent on CD22. To verify if CD22 was also required in primary murine B cells, we treated B cells from WT and CD22-KO mice with rGal9, stimulated with anti-IgM F(ab')_2_, and immunoblotted cell lysates for phospho-CD19 as described above. In contrast to our findings in human CD22-deficient Daudi B cells, galectin-9 suppression of BCR signaling was maintained in CD22-KO primary murine B cells (Supplementary Figure 1). These findings suggest differential requirements for CD22 in primary murine B cells compared to a human B cell line. The reason for this discrepancy is not clear, but may be linked to differential expression and/or glycosylation of CD45, which we identified galectin-9 binds to in primary murine B cells.

### Galectin-9 Increases Association of IgM and CD22

**Figure 2:**
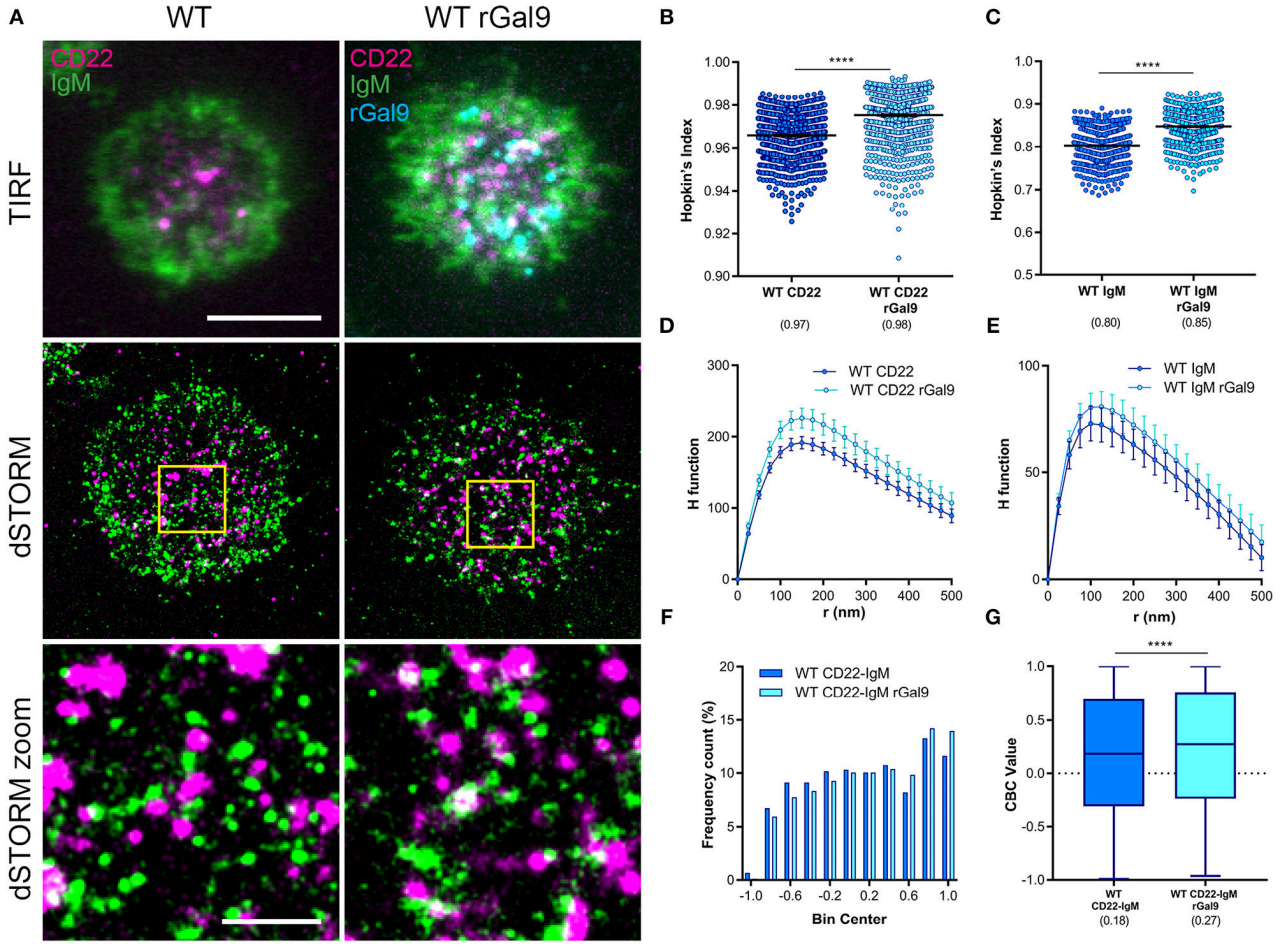
Exogenous galectin-9 increases association of IgM and CD22. **(A)** Representative merged TIRFM (top), dSTORM (middle), dSTORM zoom (bottom) images showing surface CD22 (magenta), IgM-BCR (green) on WT Daudi B cells (WT) (left) and CD22, IgM-BCR, and recombinant galectin-9 (rGal9; cyan) in rGal9 treated WT Daudi B cells (WT rGal9) (right). dSTORM ROI (3 × 3 μm) is outlined in yellow (middle) and magnified in dSTORM zoom (bottom). **(B–G)** Quantification of at least 15 ROIs from WT and WT rGal9 Daudi B cells pooled from 4 independent experiments. **(B,C)** Hopkin's index (1,000 localizations plotted) and **(D,E)** H function derived from Ripley's K for CD22 and IgM. The difference in the peak height of the H function between untreated and rGal9-treated cells was not significant by Mann Whitney. **(F)** Coordinate-based colocalization (CBC) histograms of single-molecule distribution of colocalizations between CD22 and IgM. **(G)** Box plot of CBC values of data from **(F)** showing 25–75th percentile, median, minimum, and maximum values (whiskers). Colocalization between channels shown in white. Scale bars represent 5 and 1 μm (zoom). Lines/errors represent means ± SEM. ****p < 0.0001 by Mann Whitney.

We next asked if the requirement of CD22 for galectin-9 mediated suppression of BCR signaling in human Daudi B cells was due to increased association of CD22 and IgM. We performed dual-color dSTORM of CD22 and IgM in CD22 WT Daudi B cells treated or not with rGal9. We labeled Daudi B cells with Alexa 488-labeled anti-IgM Fab fragment and Alexa 647-labeled anti-CD22 antibody and settled them on fibronectin-coated coverslips in order to adhere cells. Visual inspection of dual-dSTORM images revealed that both IgM and CD22 appear more clustered in rGal9 treated cells (Figure 2A). To quantify this observation, we examined the Hopkin's Index, which evaluates the clustering tendency compared to a random distribution, which has a value of 0.5 (28). We found that treatment with rGal9 increased the Hopkin's Index of both CD22 and IgM (Figures 2B,C). We also found a difference in the H function derived from Ripley's *K* function, which evaluates the extent of clustering (28), in rGal9-treated cells compared to untreated cells. For both CD22 and IgM, the peak height of the H function curve was increased in rGal9-treated cells, indicating an increase in the density of molecules within clusters (Figures 2D,E). Visual inspection of dual-dSTORM images also suggested an increased co-localization of IgM and CD22 in rGal9 treated cells (Figure 2A). To quantify this observation, we performed coordinate-based colocalization analysis, which ranges from +1 (perfectly colocalized) to −1 (perfectly segregated) (27). We validated our dual dSTORM imaging and image registration parameters using Daudi cells stained with Alexa Fluor 647 anti-IgM Fab and Alexa Fluor 488 anti-IgM Fab and determined the degree of colocalization. The histogram of IgM-IgM colocalization shows the percentage of molecules is highest for a CBC value of +1; ~25% of molecules being perfectly colocalized and 0% of the molecules being perfectly excluded. The median CBC value was 0.63, implying a high degree of colocalization and setting the value for the maximal level of colocalization achieved in our experiments (Supplementary Figure 2). In rGal9 treated cells, we found an increase in the frequency of positive CBC values and a concomitant decrease in the frequency of negative CBC, indicating increased colocalization of IgM and CD22 (Figure 2F). Indeed, the median CBC value in rGal9 treated cells was 0.27 compared to 0.18 for untreated cells (Figure 2G). Importantly, we obtained similar findings with respect to CD22 and IgM organization and their increased colocalization upon rGal9 treatment in parental Daudi B cells expressing endogenous CD22 (Supplementary Figure 3). These findings indicate that galectin-9 alters the organization of both CD22 and IgM-BCR nanoclusters as well as the association of CD22 and IgM-BCR, providing a mechanism for galectin-9 mediated inhibition of BCR signaling.

### Mutation of N-Glycan Sites Alters CD22 Nanoclusters

CD22 is highly clustered on the surface of naïve B cells, organized as preformed nanoclusters (29). The crystal structure of the extracellular domain of CD22 revealed that N-glycans in the outermost domains of CD22 are localized on one face and thus we hypothesized they may facilitate CD22 nanoclustering (16). To examine the role of these N-glycans in CD22 nanoclustering, we generated a CD22 mutant with point mutations of asparagine to glutamine in 5 out of the 6 N-glycans localized in the outermost domains of CD22 (denoted 5Q; Supplementary Figure 4A). The asparagine at position 101 was not mutated as this glycan is located in a hydrophobic groove between domains 1 and 2, and its mutation resulted in lack of expression (16). We generated cell lines stably expressing wild-type (WT) and the 5Q-mutant of CD22 in a Daudi B cell line in which CD22 was deleted via CRISPR-Cas9 [kindly provided by Dr. Joan Wither (Krembil Research Institute)]. Transfected B cells were sorted in order to obtain populations of WT CD22 and 5Q CD22 with similar expression to each other as well as the parental Daudi cell line prior to CRISPR-mediated deletion of CD22 (Supplementary Figures 4B,C). These cells also expressed similar levels of IgM and CD19 (Supplementary Figures 4D,E).

**Figure 3:**
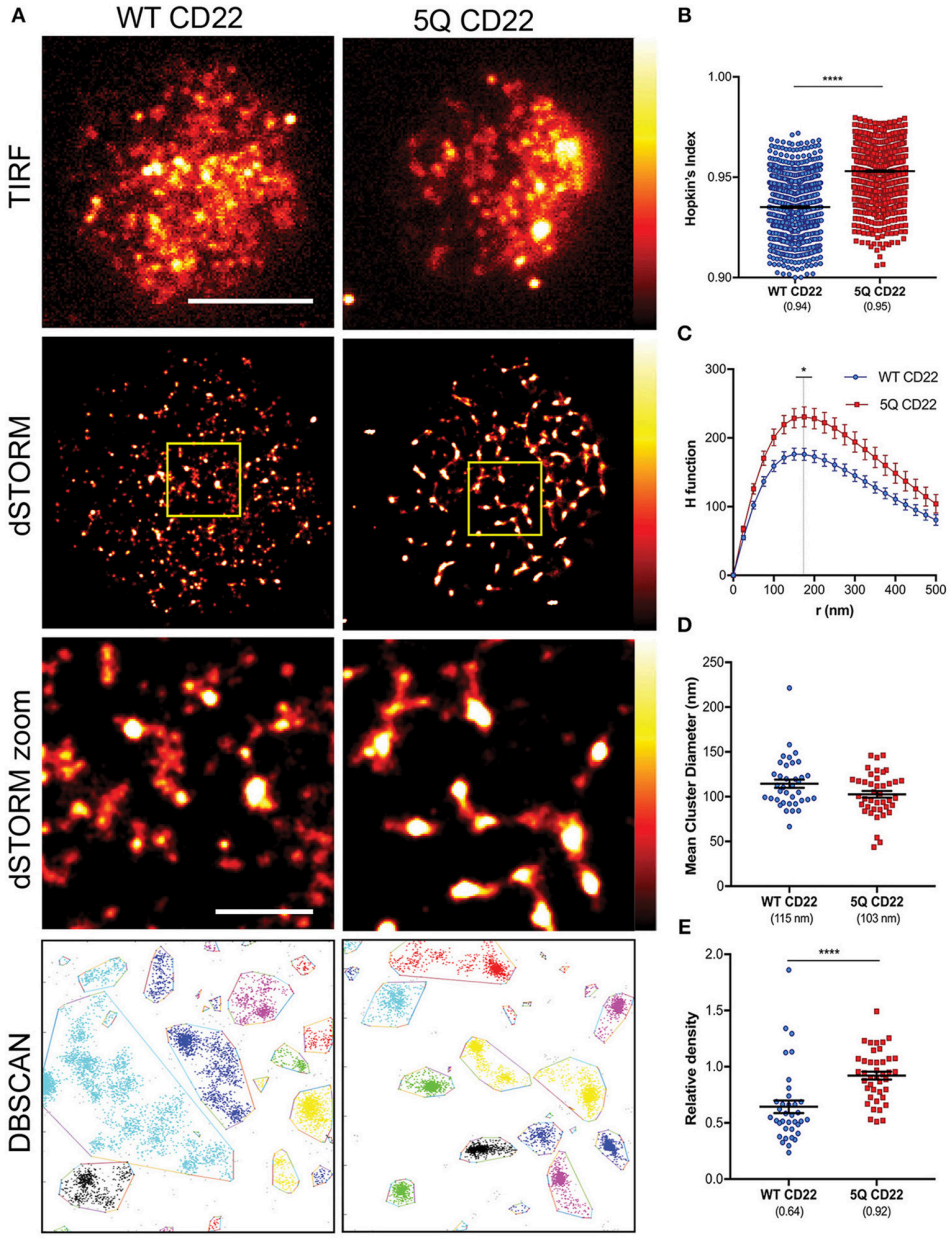
CD22 nanocluster formation is increased by mutation of N-glycan sites. **(A)** Representative TIRFM (top row), dSTORM (second row), dSTORM zoom (third row), DBSCAN clusters (bottom row) images showing surface CD22 on WT Daudi B cells (WT CD22) (left) and 5Q-mutant Daudi B cells (5Q CD22) (right). dSTORM ROI (3 × 3 μm) is outlined in yellow (second) and magnified in dSTORM zoom (third) and DBSCAN (bottom). dSTORM images are mapped to a false-color scale ranging from black to white (0–255) **(B–E)** Quantification of at least 30 ROIs from WT and 5Q-mutant Daudi B cells pooled from 4 independent experiments. **(B)** Hopkin's index (1000 localizations plotted), **(C)** H function derived from Ripley's K, **(D)** Mean diameter of CD22 clusters (1 point per ROI), and **(E)** Relative DBSCAN cluster density (1 point per ROI). Scale bars represent 5 and 1 μm (zoom). Lines/errors represent means ± SEM. *p < 0.05, ****p < 0.0001 by Mann Whitney.

To examine CD22 organization at the cell surface, WT and 5Q-mutant Daudi B cells were labeled with saturating amounts of Alexa Fluor 647 anti-CD22 whole antibody, spread on fibronectin-coated coverslips and dSTORM images acquired using total internal reflection fluorescence (TIRF) microscopy. Consistent with murine B cells (29), human CD22 is also organized in preformed nanoclusters at the cell surface (Figure 3A). Single molecule localization data was rendered as a normalized histogram, where the number of localizations corresponds to higher density. Based on the reconstructed dSTORM images, 5Q CD22 appeared to form higher density nanoclusters on the B cell surface compared to WT CD22 (Figure 3A). To quantify this observation, we again used the Hopkin's Index and the H function of Ripley's K. The mean Hopkin's Index of WT CD22 was 0.94, consistent with the clear non-random distribution of CD22 seen even in TIRF images. The Hopkin's Index of 5Q CD22 was significantly higher than WT CD22, indicating an increased tendency for clustering in the 5Q-mutant (Figure 3B). Consistent with this, the degree of clustering as measured by the peak height of H-function curve was also increased in 5Q CD22 compared to WT CD22; however, the mean cluster radius was similar at 150–175 nm (Figure 3C). To further investigate the clustering characteristics of WT and 5Q-mutant CD22, we examined the mean cluster diameter based on Getis-based clustering analysis (30, 31). This analysis method calculates a ratio between the sum of all the values within a defined distance and the sum of all the values of all the subregions, which is a measure of local clustering. Consistent with Ripley's K analysis, Getis-based clustering analysis revealed no significant difference in mean cluster diameter between WT and 5Q CD22 (Figure 3D). Finally, we also employed density-based spatial clustering of applications with noise (DBSCAN) (32). This method offers some advantages over Ripley's K analysis as it can identify arbitrarily shaped clusters, whereas Ripley's K assumes circular clusters, and is more robust to outliers and background noise (32). We employed DBSCAN analysis for insight into the relative nanocluster density, which was calculated as a ratio between number of CD22 molecules identified in a cluster and cluster diameter. Consistent with our observation of dSTORM images, CD22 cluster density is significantly increased in 5Q CD22 compared to WT CD22 (Figures 3A,E). Taken together, these data demonstrate that abrogation of N-terminal glycans on the outermost domains of CD22 increased the degree of CD22 nanoclustering and the density of molecules within nanoclusters.

### Mutation of N-Glycan Sites Does Not Alter CD22 and IgM Mobility

**Figure 4:**
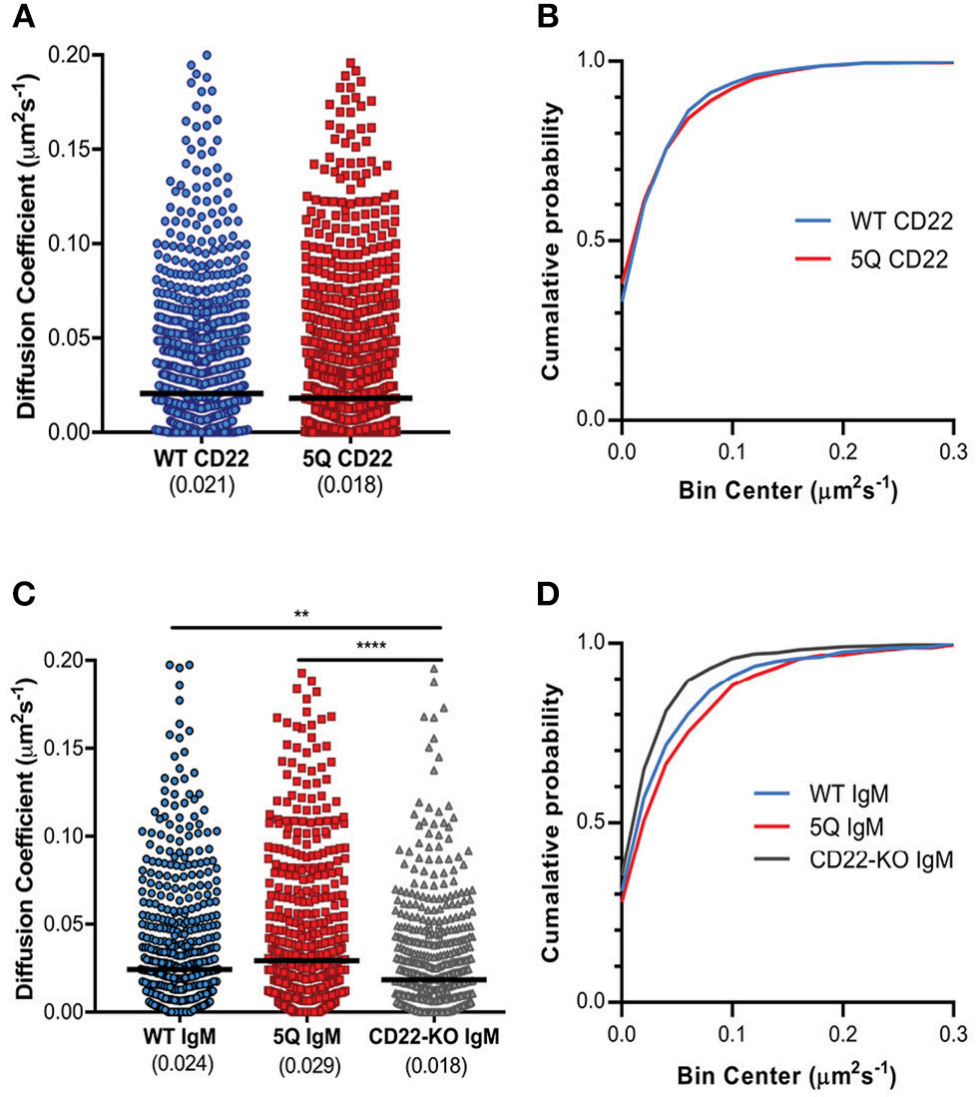
Mutation of N-glycan sites does not significantly impact CD22 mobility. **(A)** Diffusion coefficients and **(B)** cumulative probability of CD22 single molecules in WT CD22 and 5Q mutant CD22 expressing cells. **(C)** Diffusion coefficients and **(D)** cumulative probability of IgM single molecules in WT CD22, 5Q-mutant CD22, and CD22-KO cells. Quantification of at least 30 cells pooled from 4 independent experiments, 1,000 randomly selected diffusion coefficients plotted. Lines represent median. **p < 0.01, ****p < 0.0001 by Kruskal-Wallis, followed by Dunn's multiple comparisons test.

The importance of receptor mobility on function has recently been demonstrated for murine CD22, which exhibits rapid diffusion on the B cell surface, thereby providing a means of attenuating BCR signaling. Indeed, increased CD22 diffusion in the R130E mutant lacking sialic acid binding correlated with increased inhibition of BCR signaling (29). Given this, and our observation of increased CD22 nanoclustering in the 5Q-mutant, we next asked if CD22 mobility is also altered by mutation of N-glycan sites. To examine this, we performed single particle tracking analysis. WT and 5Q CD22 expressing cells were labeled with a limiting concentration of Atto-633 conjugated anti-CD22 Fab fragment for visualization of single particles by TIRF microscopy. CD22 diffusion ranged from nearly immobilized particles to highly mobile particles, similar to the heterogeneous mobility of IgM molecules (Figures 4A,B). The median diffusion coefficient of WT CD22 was 0.021 μm^2^s^−1^, while the median diffusion coefficient of 5Q CD22 was consistently, but not statistically significantly, lower (0.018 μm^2^s^−1^; Figure 4B). These data demonstrate that abrogation of these N-glycans does not significantly alter CD22 mobility.

Given that CD22 is known to interact with IgM and altering IgM mobility is sufficient to trigger BCR signaling (25), we next examined IgM mobility in WT, 5Q-mutant, and CD22-KO Daudi cells. Interestingly, the diffusion coefficient of IgM on CD22-KO Daudi B cells was significantly lower compared to IgM on WT CD22 cells (0.018 μm^2^s^−1^ compared to 0.024 μm^2^s^−1^, respectively; Figure 4C). In contrast, the diffusion coefficient of IgM on 5Q CD22 cells was significantly higher compared to IgM on CD22-deficient cells, and also higher, although not statistically significant, compared to IgM on WT CD22 cells (Figure 4C). Plotting the cumulative probability of diffusion coefficients showed that CD22-KO cells had the highest cumulative probability of diffusion coefficients in the lowest diffusion coefficient bins; whereas 5Q-mutant CD22 had a higher cumulative probability of diffusion coefficients in the higher bins (Figure 4D). Taken together, these data suggest that regardless of the existence of N-glycosylation or not, CD22 itself affects the mobility of IgM-BCR at the cell surface, possibly through CD22-IgM interactions.

### Mutation of CD22 N-Glycan Sites Enhances BCR Signaling

**Figure 5:**
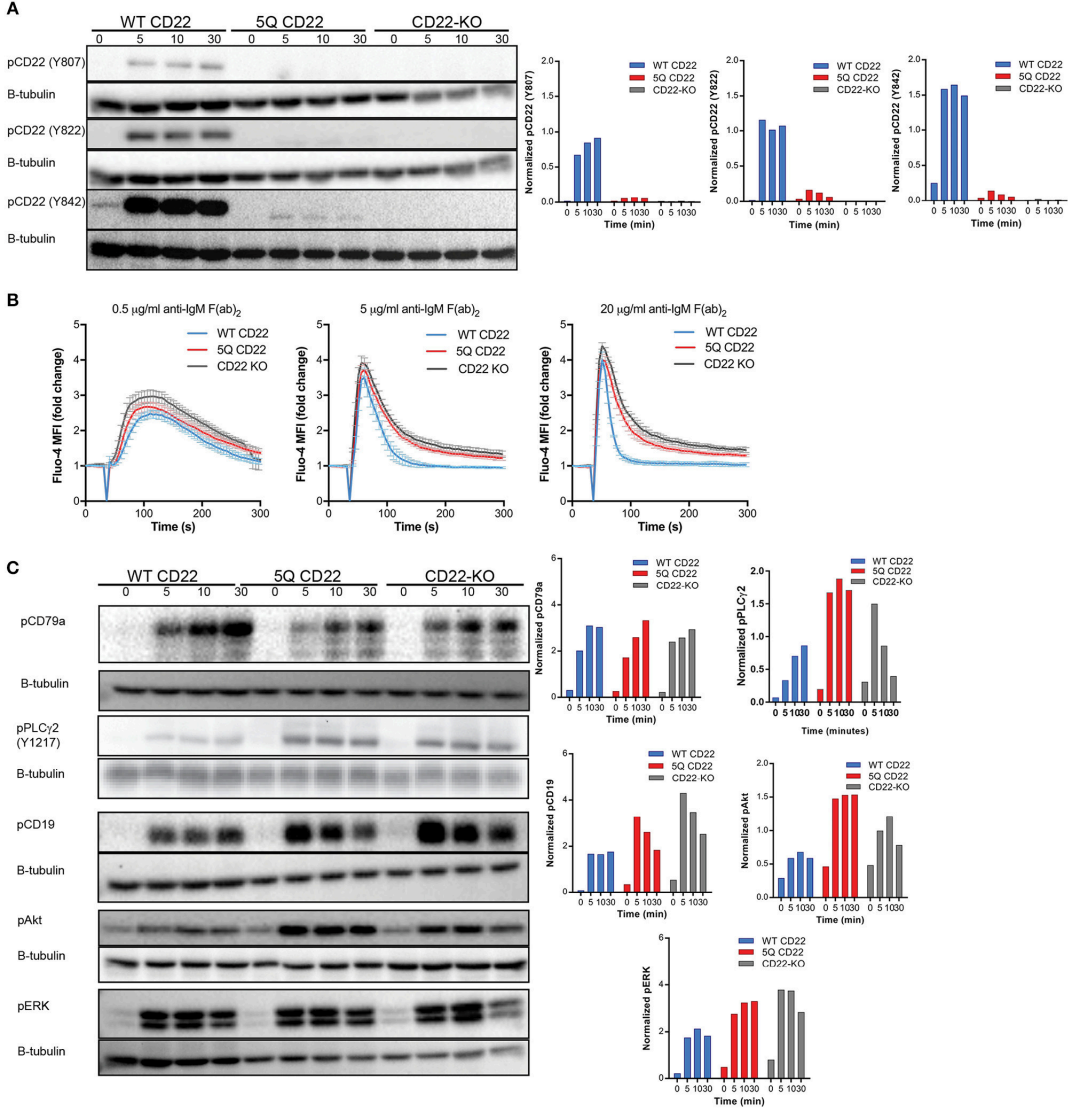
BCR signaling is enhanced by CD22 N-glycan mutation. **(A)** Representative western blots of WT (WT CD22), 5Q-mutant (5Q CD22) and CD22-KO Daudi B cells stimulated with 5 μg/ml anti-IgM F(ab')_2_ for indicated times, lysed and subjected to SDS-PAGE followed by immunoblotting for CD22 phosphorylation (pCD22) at Y807, Y822, and Y842. Blots were quantified (right) by normalizing to β-tubulin loading control. **(B)** Calcium flux induced by BCR stimulation with 0.5 μg/ml (left), 5 μg/ml (middle), and 25 μg/ml (right) anti-IgM F(ab')_2_ in WT CD22, 5Q CD22, and CD22-KO Daudi B cells. Calcium flux quantified by fold change in Fluo-4 mean fluorescence intensity over three independent experiments. **(C)** WT CD22, 5Q CD22, and CD22-KO Daudi B cells were stimulated with 5 μg/ml anti-IgM F(ab')2 for indicated times, lysed and subjected to SDS-PAGE followed by immunoblotting for phospho-CD79a, phospho-PLCγ2 (pPLCγ2), phospho-CD19 (pCD19), phospho-Akt (pAkt), and phospho-ERK1/2 (pERK). Blots were quantified (right) as above. Blots and quantification representative of least three independent experiments.

The degree of CD22 homotypic clustering correlates with CD22 function in attenuating BCR signaling, as evidenced by mutation of the sialic acid binding domain (R130E) (24, 29). In this mutant, CD22 nanoclusters are smaller, and this correlates with increased CD22 phosphorylation, increased SHP-1 recruitment, and therefore decreased BCR signaling upon stimulation (24, 29). Given our observation of altered organization of CD22 in the 5Q-mutant, we next asked if this altered spatial organization had a functional impact on CD22-mediated attenuation of BCR signaling. The cytoplasmic domain of CD22 contains six tyrosine phosphorylation sites; Y762, Y822, and Y824 are associated with ITIMs; Y807 is important for Grb2 recruitment; and the function of Y752 and Y796 have not been defined (33). To investigate the functional effect of mutation of N-glycans on CD22, we first examined CD22 phosphorylation upon BCR stimulation. WT CD22, 5Q-mutant CD22, and CD22-KO Daudi B cells were stimulated with 5 μg/ml anti-IgM F(ab')_2_ for the indicated time, lysed, and subjected to SDS-PAGE followed by immunoblotting for tyrosine phosphorylation at ITIM associated Y822 and Y842, as well as Grb2-recruitment associated Y807. Surprisingly, phosphorylation of each of these sites is severely diminished in the 5Q-mutant, comparable to CD22-deficient cells (Figure 5A). To test if mutation of N-glycan sites may have affected the structure of CD22 and rendered it non-functional, we examined phosphorylation of CD22 upon crosslinking of CD22 with an anti-CD22 antibody. We found that CD22 phosphorylation at Y822 is similar to WT CD22 (Supplementary Figure 5). Thus, alteration of N-glycans within the outermost ectodomain of CD22 severely diminishes CD22 phosphorylation upon BCR stimulation.

We next assessed how loss of CD22 phosphorylation in the 5Q-mutant upon BCR stimulation affected BCR signaling. First, we examined calcium signaling upon BCR stimulation, which is increased by loss of CD22 (10). We labeled WT, 5Q, and CD22-KO Daudi B cells with the calcium indicator, Fluo-4, and measured calcium flux upon stimulation with three different concentrations of anti-IgM F(ab')_2_ stimulation (0.5, 5, and 20 μg/ml) via flow cytometry. Consistent with lack of CD22 ITIM phosphorylation, calcium flux was increased in the 5Q-mutant of CD22 compared to WT at all concentrations tested, but most markedly at 5 and 20 μg/ml (Figure 5B). Notably, the increased calcium signaling in the 5Q-mutant was comparable with CD22-KO cells. To further interrogate BCR signaling in the 5Q-mutant, we next examined phosphorylation of both proximal and distal BCR signaling molecules. CD22 ITIM phosphorylation is integral in recruiting and activating SHP-1, which can dephosphorylated BCR associated ITAM-containing signaling chains CD79a/b (Igα/β). Thus, we first investigated the effect of mutation of CD22 N-glycan sites on phosphorylation of CD79a. WT, CD22 5Q, and CD22-KO B cells were stimulated with anti-IgM F(ab')_2_, lysed and immunoblotted with anti-phospho-CD79a. Despite the enhanced calcium response we observed, phosphorylation of CD79a was not affected in the 5Q-mutant (Figure 5C). To further investigate the pathway of BCR signaling that might be affected in the 5Q-mutant, and given that we observed increased calcium signaling, which is dependent on PLCγ2 activity, we examined phosphorylation of PLCγ2. Consistent with the enhanced calcium response we observed, phosphorylation of PLCγ2 was increased in the 5Q-mutant compared to WT cells, consistent with CD22-deficient cells (Figure 5C). CD22 ITIM phosphorylation activates SHP-1, which targets the Src family kinase Lyn, and consequently its substrates including the key BCR co-receptor CD19. Thus, we examined phosphorylation of CD19 and found that CD19 phosphorylation was enhanced in 5Q CD22 mutant cells, similar to CD22-deficient cells (Figure 5C). CD19 phosphorylation recruits signaling molecules including Vav and PI3K to amplify multiple downstream pathways. Thus, we investigated the PI3K/Akt and MAP kinase pathway by measuring Akt and ERK phosphorylation. We found enhanced phosphorylation of both Akt and ERK in 5Q CD22 mutant cells, similar to CD22-KO cells (Figure 5C). These analyses of BCR signaling reveals that abrogation of these N-glycans in the extracellular domain of CD22 significantly decreased CD22 phosphorylation, and consequently increased BCR signaling upon BCR stimulation, consistent with the BCR signaling response reported for CD22-deficient B cells (10, 34). These data demonstrate a functional importance of these N-glycan sites in CD22.

### Galectin-9 Mediated Inhibition of BCR Signaling Is Dependent on N-Glycosylation of CD22

**Figure 6:**
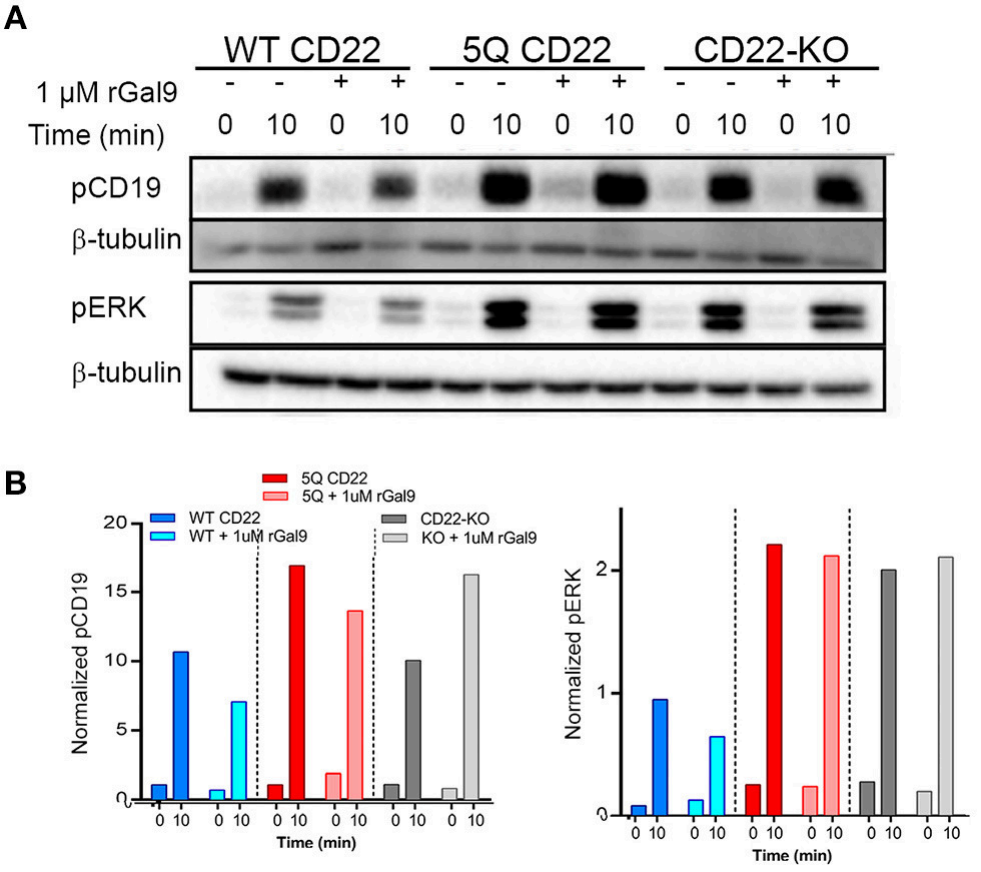
Galectin9-mediated inhibition of BCR signaling is dependent on N-glycosylation of CD22. WT (WT CD22), 5Q-mutant (5Q CD22) and CD22-KO Daudi B cells treated with 1 μM rGal9 were stimulated with 5 μg/ml anti-IgM F(ab')_2_ for indicated times, lysed and subjected to SDS-PAGE followed by immunoblotting with **(A)** anti-phospho-CD19 and anti-phospho-ERK. **(B)** Quantification of blots in **(A)** normalized to β-tubulin loading control. Blots and quantification representative of at least three independent experiments.

We identified an important role for CD22 in galectin-9 mediated inhibition of BCR signaling in human Daudi B cells. To examine if this effect was mediated by galectin-9 binding to N-glycans of CD22, we treated WT and 5Q cells with rGal9 and examined BCR signaling as described above. Of note, there is no detectable galectin-9 on the surface of cultured Daudi B cells; however, treatment with rGal9 increases surface galectin-9 in CD22 WT Daudi B cells, and to a lesser extent CD22-KO and 5Q-mutant cells, suggestive that galectin-9 may indeed bind to CD22 (Supplementary Figure 6). We found that, in contrast to WT CD22 Daudi B cells, treatment of 5Q CD22 mutant cells with rGal9 did not inhibit total tyrosine phosphorylation, nor phosphorylation of CD19 and ERK (Figure 6). These results demonstrate the importance of these N-glycan sites on CD22 for galectin-9 mediated inhibition of BCR signaling in human Daudi B cells and suggest that these sites are important for galectin-9 mediated association of CD22 with IgM.

**Figure 7:**
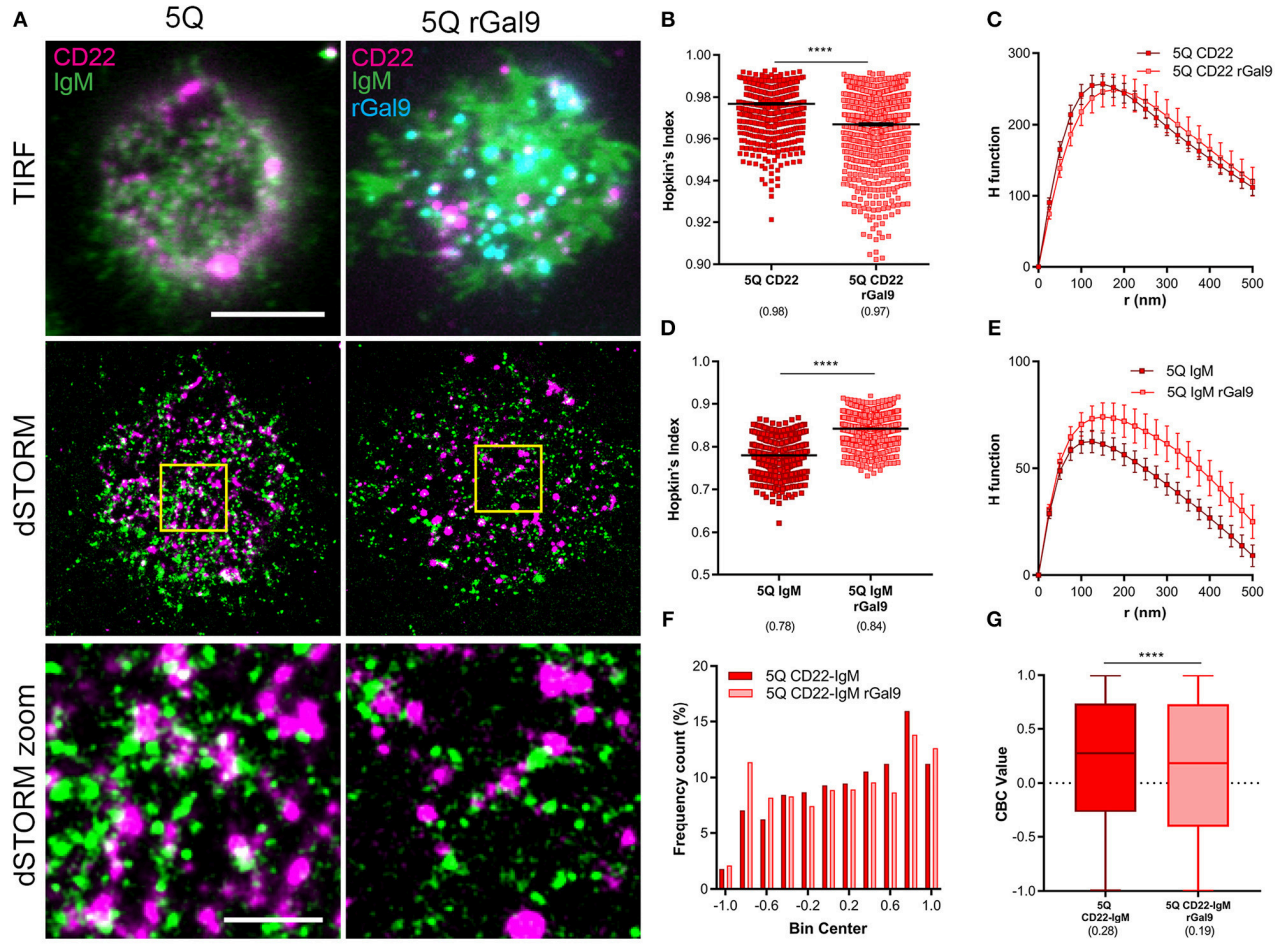
Gal9-mediated association of IgM and CD22 is dependent on N-glycans of CD22. **(A)** Representative merged TIRFM (top), dSTORM (middle), dSTORM zoom (bottom) images showing surface CD22 (magenta), IgM BCR (green), rGal9 (cyan) on 5Q-mutant Daudi B cells (5Q) (left panel) and rGal9 treated 5Q (5Q rGal9) (right panel). dSTORM ROI (3 × 3 μm) is outlined in yellow (middle) and magnified in dSTORM zoom (bottom). Colocalization between channels shown in white. Scale bars represent 5 and 1 μm (zoom). (**B–G**) Quantification of at least 15 ROIs from 5Q and 5Q + rGal9 Daudi B cells pooled from 4 independent experiments. **(B)** Hopkin's index, and **(C)** H function derived from Ripley's K for CD22. **(D)** Hopkin's index and **(E)** H function for IgM. Lines/errors represent means ± SEM. **(F)** Coordinate-based colocalization (CBC) histograms of single-molecule disributions of colocalization between CD22 and IgM. **(G)** Box plot of CBC values of data from **(F)** showing 25–75th percentile, median, minimum, and maximum values (whiskers). ****p < 0.0001 by Mann Whitney.

To examine this, we performed dual-dSTORM to assess the impact of rGal9 on the organization and association of IgM and CD22 in 5Q CD22 mutant expressing cells (Figure 7A). Dual dSTORM analysis of the clustering tendency of CD22 and IgM was evaluated by Hopkin's index and Ripley's K H function. We found that, in contrast to WT CD22, which exhibits increased clustering upon rGal9 treatment (Figure 2), this treatment decreased CD22 clustering tendency in the 5Q-mutant (Figure 7B). Ripley's K analysis showed a small right shift in the peak of the H function curve, suggesting a small increase in the size of CD22 nanoclusters in the 5Q-mutant, but without a significant change in the relative density of molecules within clusters (Figure 7C). In contrast, treatment with rGal9 increased the clustering tendency of IgM in 5Q-mutant cells (Figure 7D), similarly to that observed in WT cells (Figure 2C). This increased clustering was also reflected in the H-function curve which showed an increase in the peak as well as an increase across all r-values (Figure 7E), similar to WT cells treated with rGal9 (Figure 2E). Visual inspection of dual dSTORM images suggested that treatment with rGal9 decreased the association of IgM and CD22 in the 5Q-mutant compared to untreated cells (Figure 7A). Indeed, CBC analysis demonstrated that the frequency of negative CBC values increased and the frequency of positive values decreased (Figure 7F). The median CBC value of CD22-IgM colocalization decreased from 0.28 in untreated 5Q CD22 cells to 0.19 in rGal9 treated cells (Figure 7G). Together, these results demonstrate that treatment with rGal9 segregates IgM and CD22 in 5Q CD22 expressing cells. Taken together, these findings indicate that mutation of these five N-glycan sites on CD22 alter both CD22 and IgM organization in the steady-state, as well as galectin-9 mediated clustering of CD22, which consequently impacts galectin-9 mediated inhibition of BCR signaling.

## Discussion

**Figure 8:**
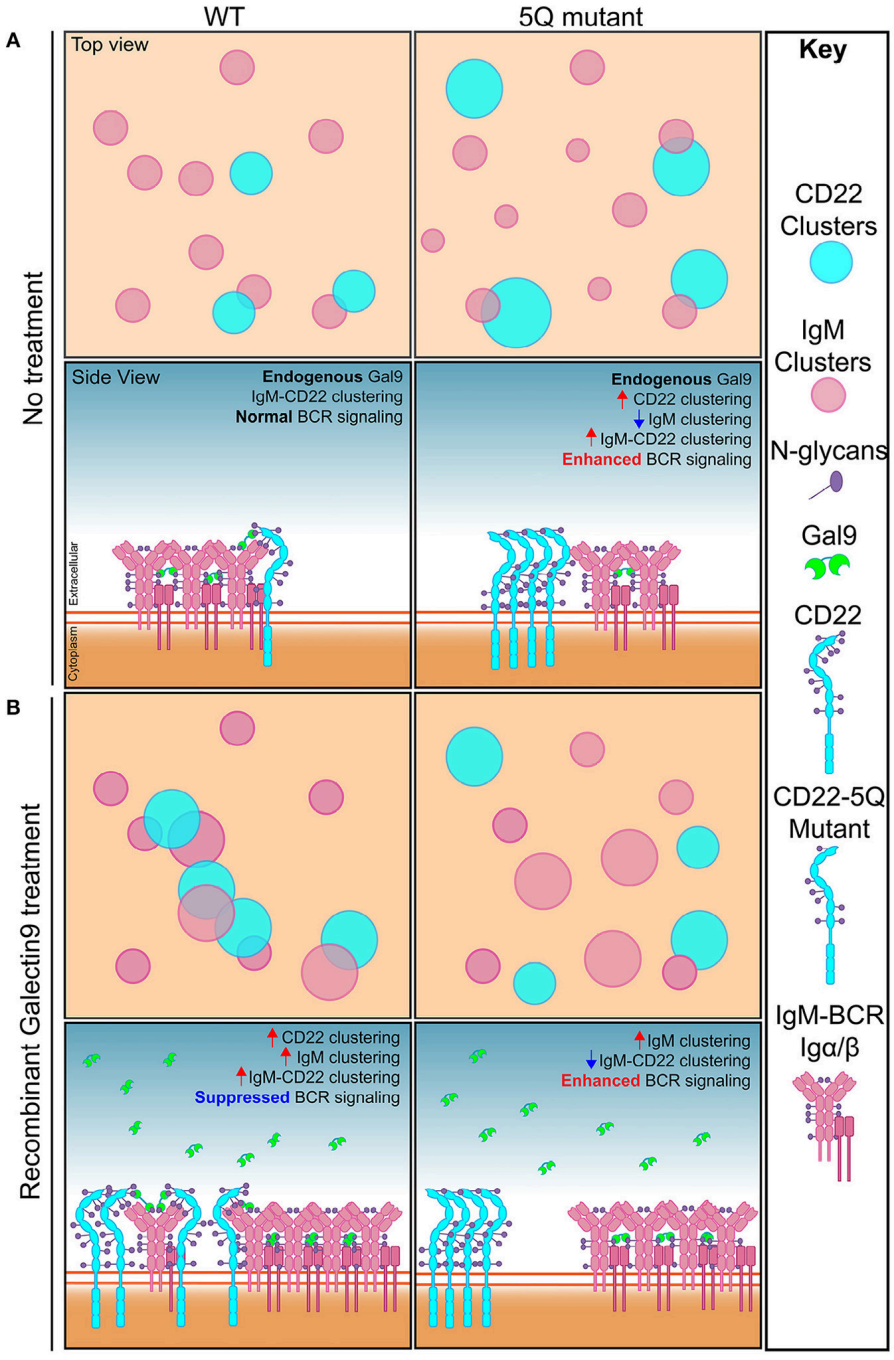
Model depicting how N-glycosylation regulates CD22 organization and function. **(A)** Top and side view of IgM and CD22 organization on the surface of resting WT B cells (left) and 5Q-mutant Daudi B cells (right). In resting WT B cells, galectin-9 mediates basal interaction between IgM and CD22 to allow for regulated BCR signaling. Mutation of five N-linked glycosylation sites from asparagine to glutamine on CD22 leads to increased CD22 clustering, decreased IgM clustering, decreased CD22 phosphorylation, and increased BCR signaling. **(B)** Top and side view of IgM and CD22 organization on the surface of recombinant galectin-9 (rGal9) treated WT (left) and 5Q-mutant (right) B cells. In resting WT B cells, treatment with rGal9 increases IgM and CD22 nanoclusters and increases IgM-CD22 colocalization leading to suppression of BCR signaling upon antigen stimulation. Treatment of 5Q-mutant with rGal9 increases IgM cluster size and decreases IgM-CD22 colocalization. rGal9 treatment does not reduce BCR signaling in 5Q-mutant B cells as observed in WT B cells, demonstrating that galectin-9 mediated regulation of BCR signaling is dependent on CD22 N-glycans.

Here, we used super-resolution imaging and biochemical assays to identify that N-glycans in the ectodomain of CD22 are important for CD22 organization and function, through a mechanism involving the secreted lectin, galectin-9. We propose the following model to account for our data: in WT cells, under steady-state conditions, galectin-9 binds to IgM and CD22 mediating close association of these molecules, or nanoclusters of molecules, and propose that this interaction may help set the threshold of B cell activation. Treating WT cells with rGal9 increases CD22-IgM co-localization/co-clustering and consequently reduces BCR signaling. Mutation of 5 N-glycans in the outmost ectodomains of CD22 increases the density of CD22 nanoclusters, and increases CD22-IgM co-clustering; however, despite this increased co-localization, CD22 phosphorylation upon BCR stimulation is decreased, and consequently BCR signaling is enhanced. Treatment of these cells with rGal9 decreases CD22-IgM co-clustering, which may be due to enhanced clustering of IgM in this context, but nevertheless, galectin-9 mediated inhibition of BCR signaling is abrogated (Figure 8). These findings identify a novel non-sialic acid binding mechanism regulating CD22 organization and function.

CD22 has been shown to associate with other CD22 molecules via a sialic acid binding dependent mechanism to form discrete nanoclusters (15, 29). Recently, the crystal structure of CD22 demonstrated that five N-glycan sites are located on one face of the protein in the first two N-terminal domains, and were hypothesized to be involved in CD22 homotypic interactions via sialic acid binding based on their position (16). Instead, we found that ablation of these N-glycan sites increased the degree and density of CD22 nanoclusters compared to WT CD22. Based on experimental design, we cannot rule out that a terminally sialylated glycan at asparagine 101, which was not mutated due to lack of CD22 expression, may act as a *cis* ligand for CD22 on the B cell surface. In this case, the increased clustering of CD22 observed in the 5Q-mutant may be due to lack of steric hindrance around the sialic acid binding domain allowing for increased binding to terminal sialic acid at N101 of neighboring CD22 molecules. Conversely, increased nanocluster density may also be due to a role for these N-glycans in mediating CD22 heterotypic interactions with other cell surface proteins, lack of which may increase homotypic CD22 clustering. Indeed, we hypothesized that these N-glycans may mediate heterotypic interactions via indirect protein-protein interactions with IgM that is independent of CD22 sialic acid binding, but instead mediated by the soluble secreted lectin, galectin-9, based on our recent study demonstrating that galectin-9 binds to IgM-BCR and regulates its organization, mobility, and consequently activation upon antigen stimulation (6).

Here, we demonstrate using CD22-deficient human Daudi B cells that galectin-9 mediated inhibition of BCR signaling requires CD22, as hypothesized in our previous working model. We also now demonstrate using dual-color super-resolution imaging that galectin-9 increases the association of IgM and CD22. This increased association is abrogated upon mutation of five N-glycan sites in the outermost ectodomains of CD22, and corresponds to loss of galectin-9 mediated inhibition of BCR signaling. While it is not clear why abrogation of N-glycan sites on CD22 increased co-localization between IgM and CD22 in the steady-state, one possibility is that the more randomly distributed IgM molecules in the 5Q-mutant may be in closer vicinity to higher density CD22 clusters independent of galectin-9. These findings suggest that galectin-9 may mediate IgM-CD22 organization through one of two mechanisms. Galectin-9 may bind directly to CD22 via one or more of these N-glycan sites to mediate association with IgM-BCR. Alternatively, we identified CD45 as a ligand of galectin-9 (6), and so it may be that these CD22 N-glycans impact heterotypic interactions between CD22 and CD45, and it is CD45 that binds galectin-9 to localize CD22 with BCR. Regardless, our findings highlight the importance of CD22 N-glycans in galectin-9 mediated association of IgM and CD22 and the corresponding regulation of BCR signaling.

Our findings raise several points of note not only with respect to B cell biology but more broadly in terms of the functional relevance of protein nanoclusters. In recent years, with the advent of high resolution imaging modalities, there has been much interest in defining the spatial organization of cell surface proteins (35). Although still contentious with respect to some cell surface proteins (36), it appears that nanoclustering is a defining feature of most cell surface proteins (37). On the other hand, we are only beginning to understand the functional significance of nanoclusters. Constitutively clustering may, as proposed for major histocompatibility (MHC) class I and II, enhance antigen presentation and thus facilitate T cell receptor recognition of rare peptide-MHC complexes (38, 39). Clustering of proteins may also facilitate the segregation of functional units of signaling complexes, such as IgM-BCR and the co-receptor CD19 (40). Our findings contribute to this body of understanding by demonstrating altered nanocluster organization is associated with a functional defect in CD22 activity. So, how would high-density nanoclusters in the 5Q-mutant have a direct influence on lack of CD22 phosphorylation in the intracellular domain upon BCR stimulation? One possibility is that increased density of CD22 nanoclusters may be analogous to auto-inhibitory BCR oligomers as proposed by the dissociation activation model (41). In this model, the ITAMs of Igα/Igβ are inaccessible for phosphorylation by Lyn in high-density BCR oligomers in the steady-state. Alternatively (or additionally), the lipid environment around high-density CD22 nanoclusters may be altered and consequently Lyn, the Src family kinase that phosphorylates CD22 is excluded. Indeed, treatment of B cells with a synthetic high affinity ligand for CD22 prevents antigen-induced CD22 re-localization to lipid rafts, where active Lyn is localized, and consequently lack of CD22 phosphorylation (42). Moreover, we identified that galectin-9 relocalizes CD22 to Lyn-rich lipid raft domains (6), supporting the idea that these N-glycans are important for regulating the partitioning of CD22 into different domains on the B cell membrane. Examining the localization of CD22 relative to lipid raft domains, and the impact of altered N-linked glycosylation is an important future direction.

It may also be that altered heterotypic interactions, specifically with IgM-BCR, result in lack of CD22 phosphorylation in the glycan mutant. Previous studies have shown that the ability of CD22 to associate with IgM upon antigen stimulation corresponds to increased CD22 phosphorylation and greater attenuation of BCR induced calcium flux (24, 29, 43, 44). These studies indicate that disruption of CD22 homodimers and nanoclusters allows for more CD22 to be available to interact with IgM, and consequently stronger inhibition of BCR signaling. Our findings that treatment of WT B cells with exogenous galectin-9 increases heterotypic interactions between IgM and CD22, and concomitant suppression of BCR signaling upon antigen stimulation is consistent with these studies. Our results are also consistent as we observed increased CD22 nanoclustering in the 5Q-mutant and this correlates with decreased CD22 phosphorylation and increased BCR signaling. Although our steady-state analysis of the glycan mutant CD22 revealed increased CD22-IgM colocalization by dual dSTORM, we hypothesize that the increased density of CD22 in larger nanoclusters and the decreased clustering of IgM results in lack of coordinated IgM-CD22 organization upon BCR stimulation, and consequently lack of CD22 phosphorylation and increased BCR signaling.

Another interesting finding of our study is that, despite markedly altered CD22 nanoclustering in the 5Q-mutant, we did not observe any significant difference in the cell surface mobility of CD22. We also hypothesized increased IgM mobility in CD22-deficient cells due to lack of CD22-IgM interactions. In contrast, we found that IgM mobility was decreased in CD22-deficient cells. It may be that CD22-IgM interactions make up a pool of highly mobile IgM molecules, or that lack of CD22-IgM interactions results in stronger association of IgM with less mobile molecules, for example, CD45. If CD22 N-glycans play a role in CD22-IgM interactions then we would expect that mutation of these sites would also result in decreased IgM mobility; however this was not observed. IgM mobility on 5Q CD22 was significantly higher compared to IgM on CD22-KO B cells. Interestingly, the mobility of IgM was not significantly altered in neither CD45-KO B cells nor sialic acid binding mutant CD22-R130E B cells, both of which altered CD22 mobility (29). However, these studies used primary murine cells and high affinity ligands differ between murine and human CD22. Our SPT data of CD22 and IgM mobility does imply altered CD22-IgM interactions given the altered IgM mobility; however, the mechanism by which N-terminal glycans and CD22 itself alter IgM mobility is not evidently conclusive.

Our results highlight a novel role for N-terminal glycans on CD22 nanocluster formation and function in attenuating BCR signaling. Importantly, lack of glycosylation at five sites in the extracellular domain results in lack of CD22 phosphorylation, and increased B cell signaling similar to CD22 deficient cells. We further demonstrate that these N-glycans are important in galectin-9 mediated inhibition of BCR signaling. While CD22 sialic acid binding mediates CD22 homotypic nanocluster formation, our results demonstrate a novel role for galectin-9 mediated CD22 heterotypic interaction with IgM dependent on CD22 N-terminal glycans. We currently do not know how the degree of CD22 glycosylation and terminal sialylation creating CD22 homotypic *cis* ligands varies in different B cell subsets and how it may be altered in B cell diseases such as cancer and autoimmunity. Future studies should assess the role of each of these glycan sites individually in altering CD22 organization and function and characterize glycosylation at these sites in B-cell pathologies. Our findings highlight the importance of CD22 glycan sites in regulating BCR signaling thresholds and that investigation of CD22 N-glycans may provide novel insight into the mechanism of altered B cell activation in disease.

## Data Availability

The raw data supporting the conclusions of this manuscript will be made available by the authors, without undue reservation, to any qualified researcher.

## Author Contributions

LW, FHMB, and BT designed the study. LW, FHMB, MY, and BT conducted experiments and data analysis. TS and JE-O provided critical material to carry out the research. J-PJ helped conceive the research. LW, FHMB, and BT wrote the manuscript, and all authors commented on it.

### Conflict of Interest Statement

The authors declare that the research was conducted in the absence of any commercial or financial relationships that could be construed as a potential conflict of interest.
