# Supplementary figures and images for: N-Linked Glycosylation Regulates CD22 Organization and Function
Source: Front Immunol. 2019 Apr 4;10:699. doi: 10.3389/fimmu.2019.00699 (PMC6458307; doi:10.3389/fimmu.2019.00699)

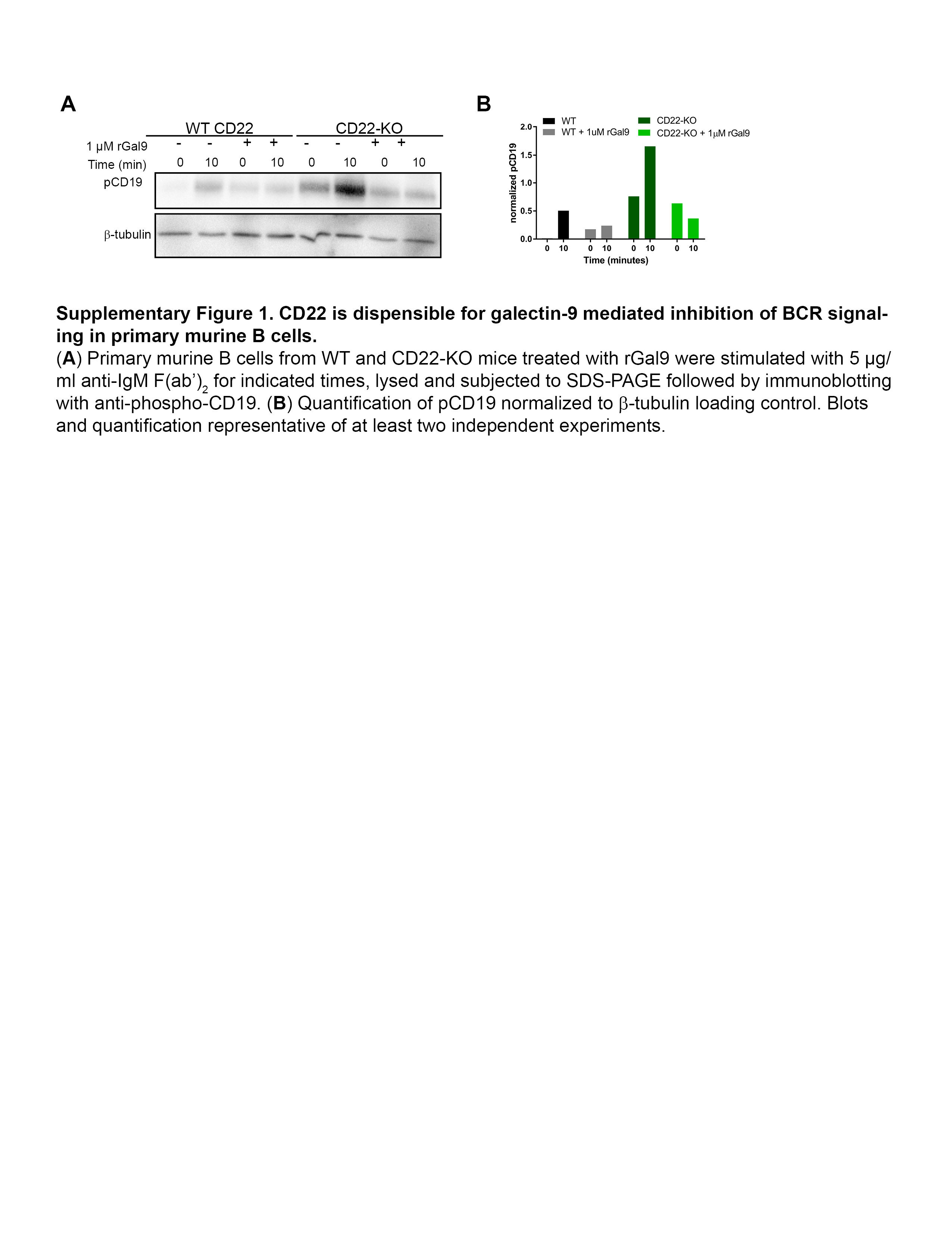

Supplement: Supplementary file 1 [file Image_1.JPEG]

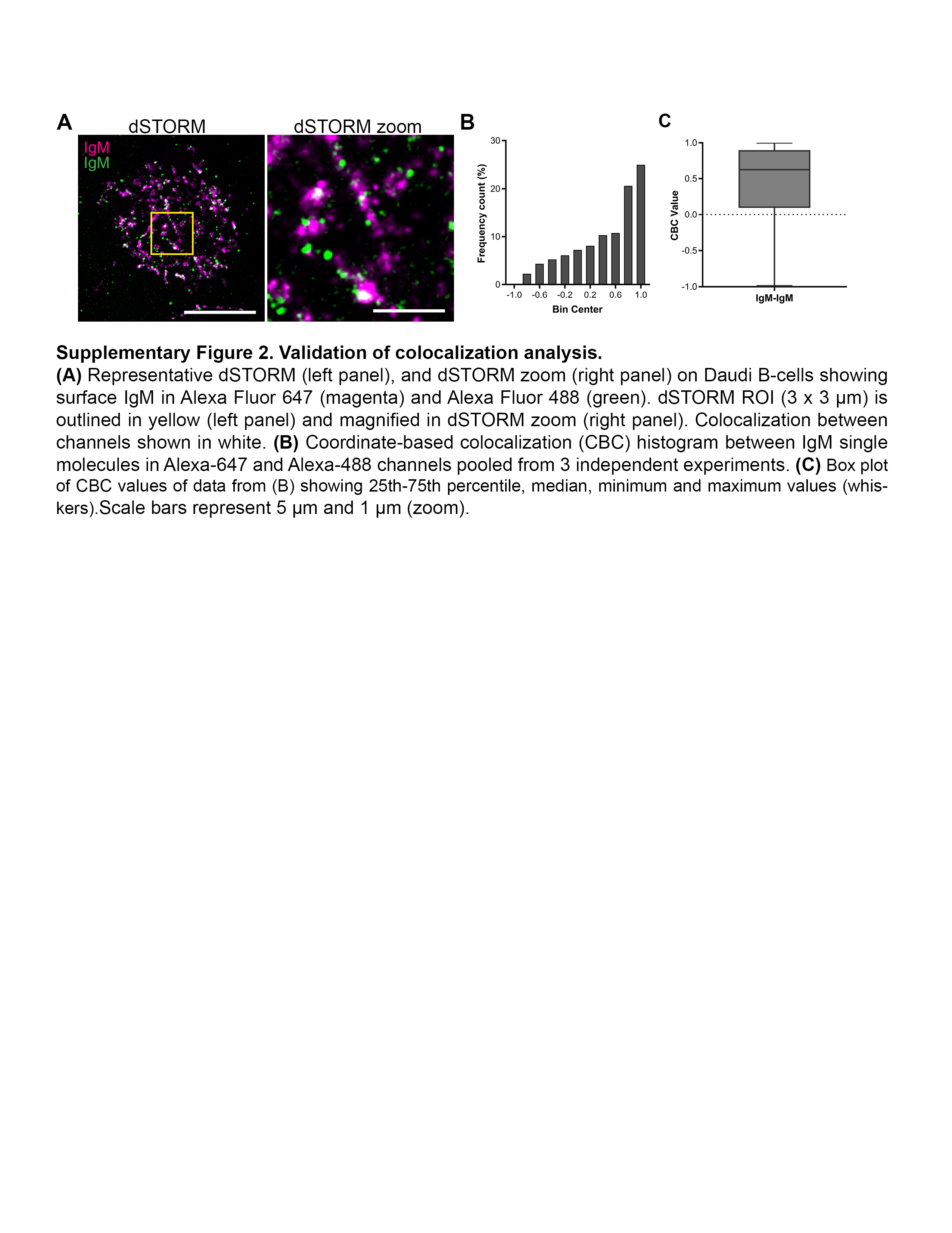

Supplement: Supplementary file 2 [file Image_2.JPEG]

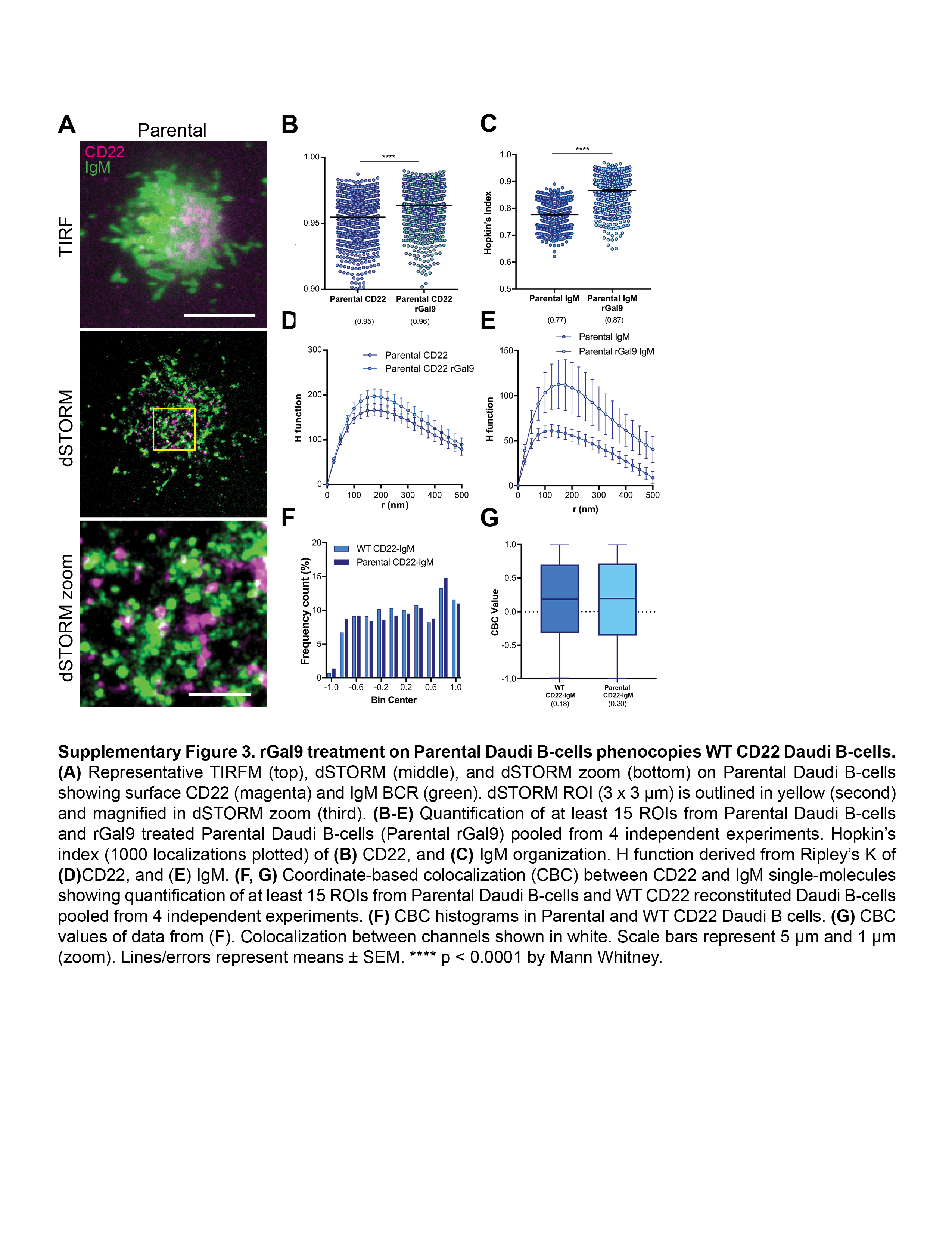

Supplement: Supplementary file 3 [file Image_3.JPEG]

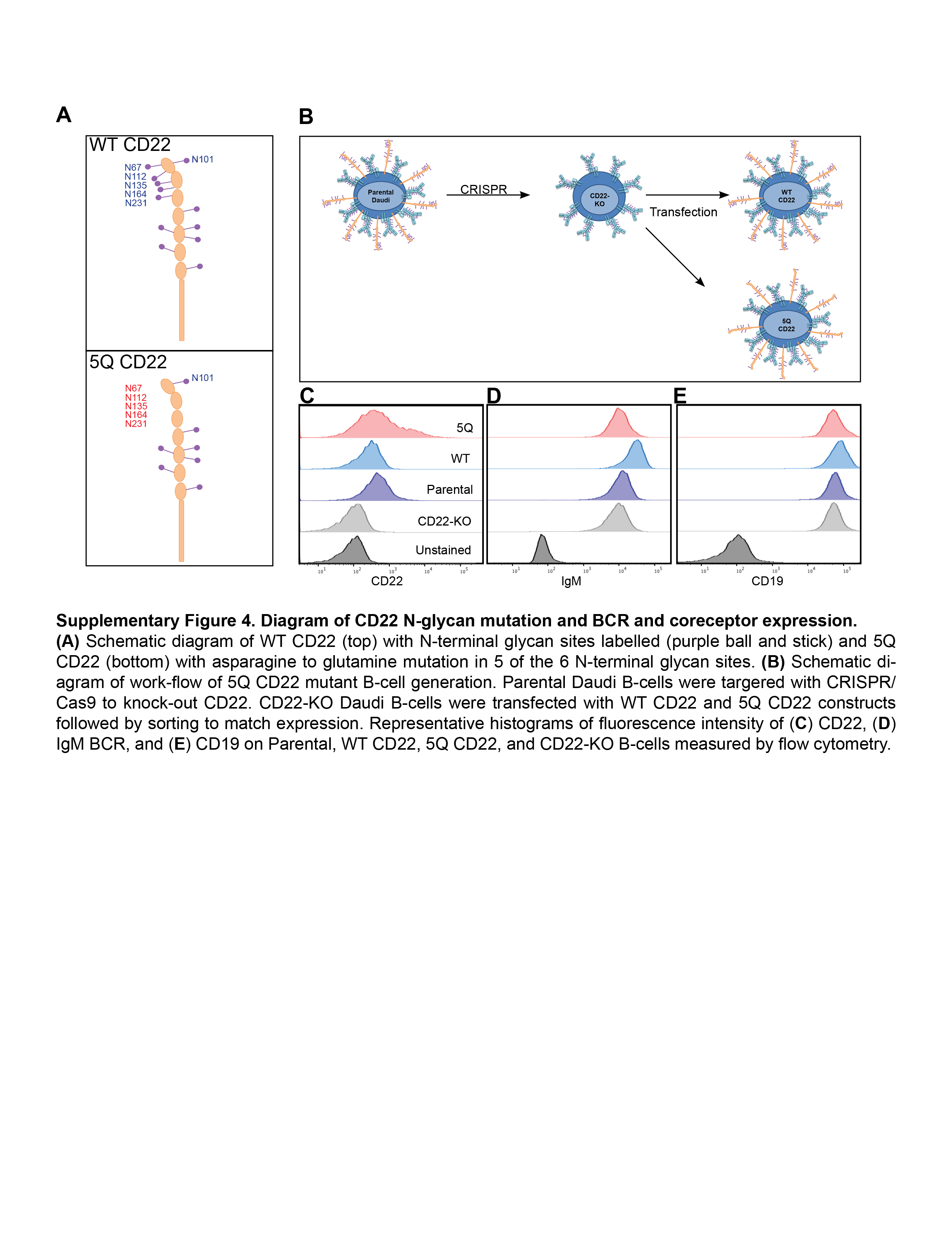

Supplement: Supplementary file 4 [file Image_4.JPEG]

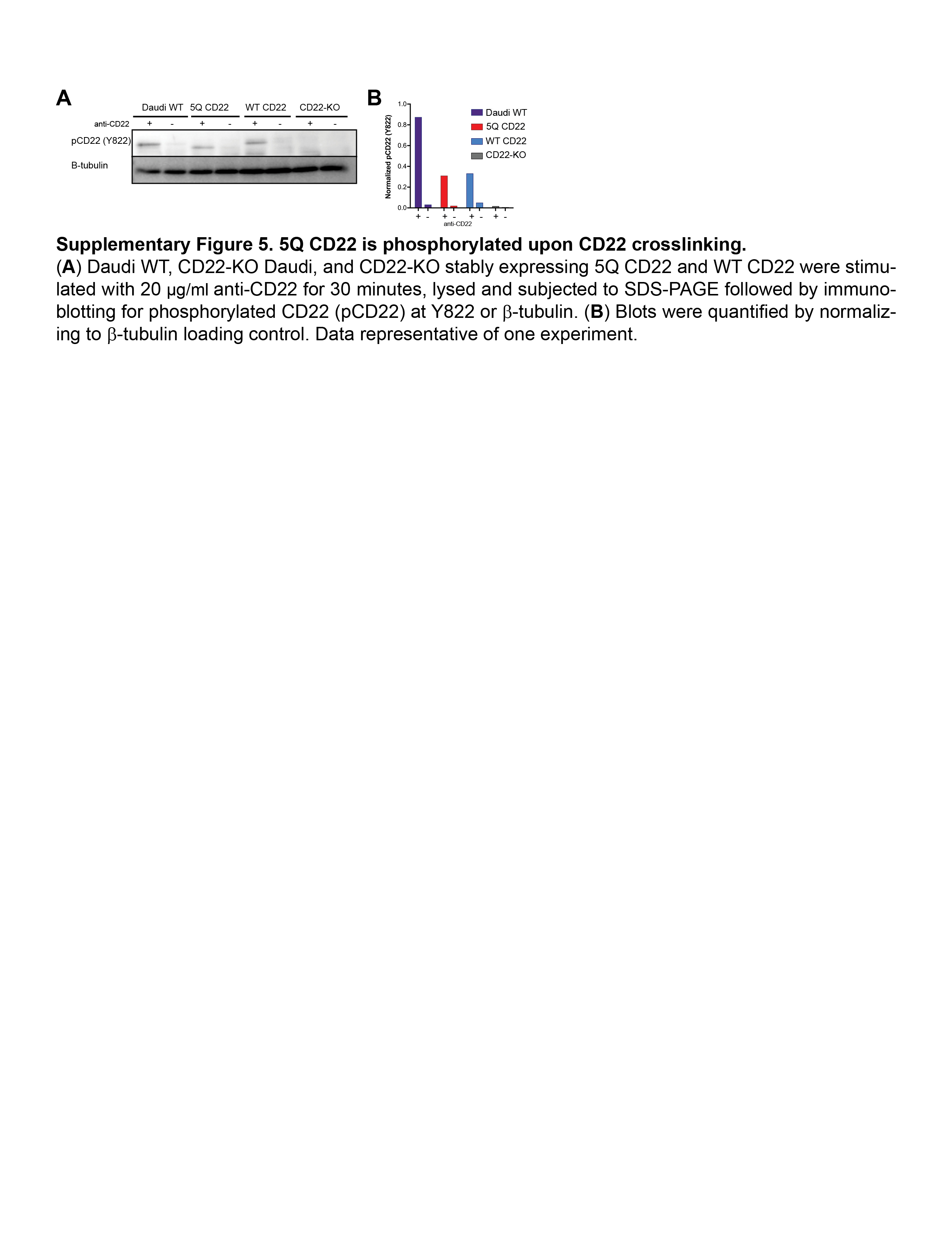

Supplement: Supplementary file 5 [file Image_5.JPEG]

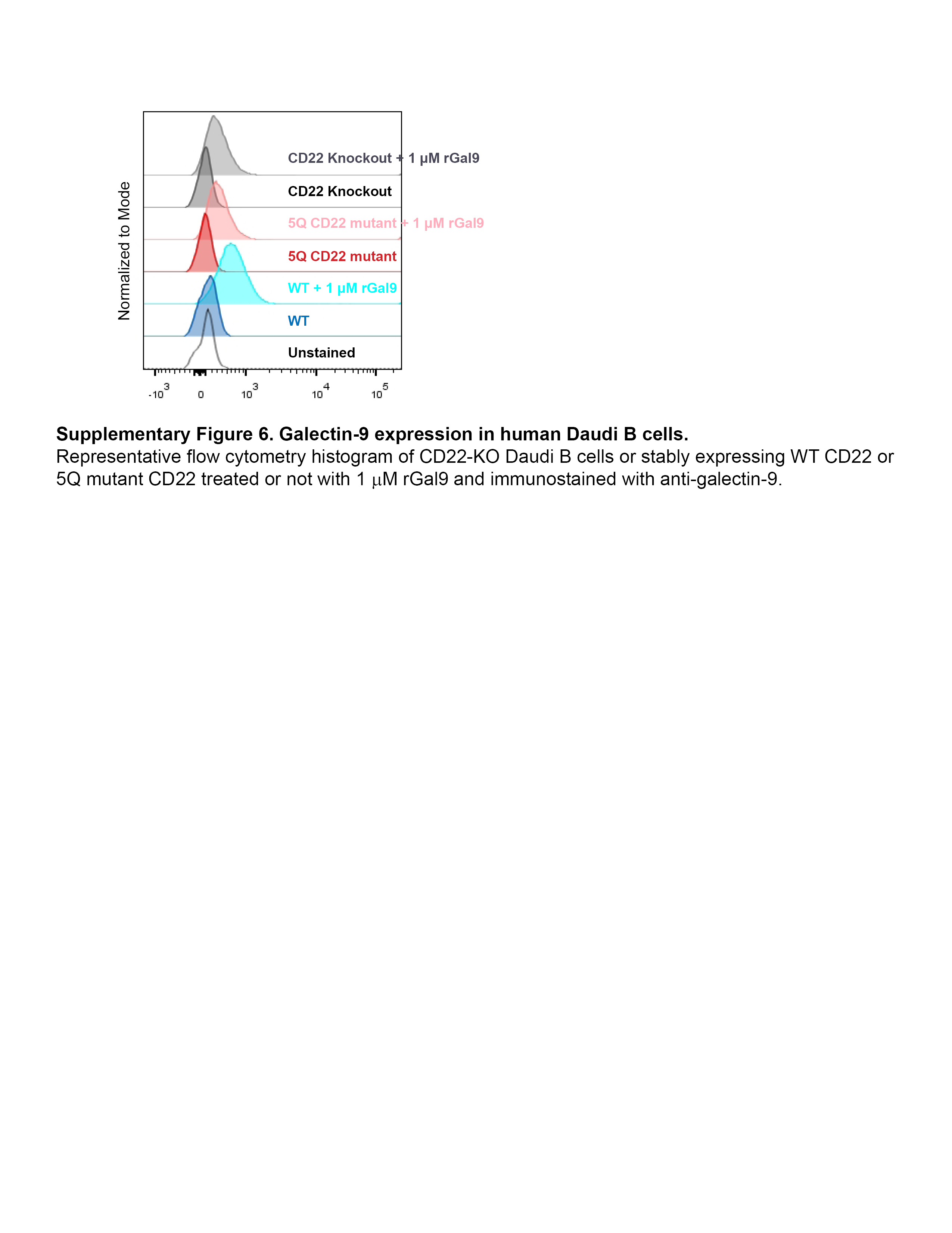

Supplement: Supplementary file 6 [file Image_6.JPEG]
